# Bayesian model selection for COVID-19 pandemic state estimation using extended Kalman filters: Case study for Saudi Arabia

**DOI:** 10.1371/journal.pgph.0003467

**Published:** 2024-07-25

**Authors:** Lamia Alyami, Saptarshi Das, Stuart Townley

**Affiliations:** 1 Centre for Environmental Mathematics, Faculty of Environment, Science and Economy, University of Exeter, Penryn Campus, Penryn, United Kingdom; 2 Department of Mathematics, College of Science, Najran University, Najran, Saudi Arabia; 3 Institute for Data Science and Artificial Intelligence, University of Exeter, Exeter, Devon, United Kingdom; 4 Environment and Sustainability Institute, University of Exeter, Penryn Campus, Penryn, Cornwall, United Kingdom; Mahidol University, THAILAND

## Abstract

Quantifying the uncertainty in data-driven mechanistic models is fundamental in public health applications. COVID-19 is a complex disease that had a significant impact on global health and economies. Several mathematical models were used to understand the complexity of the transmission dynamics under different hypotheses to support the decision-making for disease management. This paper highlights various scenarios of a 6D epidemiological model known as SEIQRD (Susceptible-Exposed-Infected-Quarantined-Recovered-Deceased) to evaluate its effectiveness in prediction and state estimation during the spread of COVID-19 pandemic. Then we investigate the suitability of the classical 4D epidemiological model known as SIRD (Susceptible-Infected-Recovered-Deceased) in the long-term behaviour in order to make a comparison between these models. The primary aim of this paper is to establish a foundational basis for the validity and epidemiological model comparisons in long-term behaviour which may help identify the degree of model complexity that is required based on two approaches viz. the Bayesian inference employing the nested sampling algorithm and recursive state estimation utilizing the Extended Kalman Filter (EKF). Our approach acknowledges the potential imperfections and uncertainties inherent in compartmental epidemiological models. By integrating our proposed methodology, these models can consistently generate predictions closely aligned with the observed data on active cases and deaths. This framework, implemented within the EKF algorithm, offers a robust tool for addressing future, unknown pandemics. Moreover, we present a systematic methodology for time-varying parameter estimation along with uncertainty quantification using Saudi Arabia COVID-19 data and obtain the credible confidence intervals of the epidemiological nonlinear dynamical system model parameters.

## 1 Introduction

Throughout history, epidemics had an impact on societies, with different forms and levels of severity. The most recent among these is the COVID-19 pandemic which is classified under the Coronaviridae family. The COVID-19 pandemic caused by severe acute respiratory syndrome coronavirus-2 (SARS-CoV-2) has caused substantial disruptions in normal public life on a global scale [1]. Studying the behaviour of this pandemic from various perspectives and methodologies has been carried out through the largest collaboration of public health professionals, mathematical modellers and data analysts who have dedicated their research to understanding the severity of this virus and its new variants. These studies were constructive for forecasting national and regional medical aids like hospital capacities, critical care, advice on economic status in business, controlling the spread, and finding suitable therapeutics and non-pharmaceutical interventions. Since data is not always adequate and accurate to understand complex systems practically in the epidemiology field. Then, connecting the available data with mathematical models helps understand the behaviour of the transmission, test parameters sensitivity, plan for the future, implement the interventions and allocate resources accordingly.

Modelling any infectious disease system should be as simple as possible and as complex as needed. There have been numerous epidemiological models with different compartments depending on the research aims in epidemiology. Despite the World Health Organization (WHO) declaring an end to the global health emergency of the COVID-19 pandemic, many individuals continue to experience COVID-19 symptoms. It remains imperative to adopt proactive measures based on insights gleaned from COVID-19 studies and modelling to mitigate the risk of future pandemics [2]. The fundamental epidemiological model called the SIR model, which divides the population into three compartments (Susceptible-Infected-Recovered), was introduced by Kermack and McKendrick in 1927, as presented in [3]. This model describes the dynamics of an infectious disease and how the number of individuals in each compartment changes over time. In the context of the COVID-19 pandemic, the SIR model has been utilised widely to predict the COVID-19 outbreak such as [4–6]. However, in many scenarios, the dynamics of infectious disease spread become very complex, and the SIR model needs to be expanded. This resulted in researchers extending the SIR model to different compartments. Several modifications study the dynamics of COVID-19 transmission, such as the SIRD model (Susceptible-Infected-Recovered-Deceased) [7, 8], SEIRD (Susceptible-Exposed-Infected-Recovered-Deceased) [9], SEIQR model (Susceptible-Exposed-Infected-Quarantine-Recovered) in [10, 11], SEIQRD model (Susceptible-Exposed-Infected-Quarantine-Recovered-Deceased) [12, 13] and other classes of models under the SIR framework. Among the various models available in the literature, the SEIQRD model has been chosen for the analysis of COVID-19 in this paper for the following reasons:

- The SEIQRD model has a clear structure, extending the SIR model by incorporating compartments for susceptible, exposed, infected, quarantined, recovered, and deceased individuals.
- The SEIQRD model aims for a balance between complexity and simplicity, considering potential challenges associated with over-parameterisation. This ensures the interpretability of model outputs, making it suitable for this study.
- Parameters selection in this model are essential and meaningful for describing the dynamics of COVID-19, focusing on key epidemiological factors such as transmission rates, incubation period, quarantine rate, recovery rates, and death rate. This approach avoids overwhelming the analysis with unnecessary complexity.
- The SEIQRD model can be extended easily by incorporating additional parameters, as introduced in this paper.
- The SEIQRD model aligns with the real data used in this study, particularly in terms of modelling active cases and cumulative death cases.
- The SEIQRD model captures the essential dynamics of COVID-19, addressing different stages of this pandemic.
- In terms of parameter sensitivity, it is evaluated and simulated under different interventions, with plausible trajectories.

One of the methods employed in estimating the COVID-19 pandemic is the Kalman filter algorithm, utilized for enhancing prediction accuracy. The well-known Kalman filter (KF) algorithm proposed by R.E. Kalman in 1960 [14], is a standard method for state estimation problems and recursive tracking of a system’s states over time. This approach is commonly employed in prediction and state estimation, effectively addressing uncertainties inherent in measured data. The popularity of the KF algorithm lies in its ability to minimize the error associated with the estimation processes. By iteratively incorporating new measurements and adjusting predictions. It dynamically adapts to uncertainties in the system and the measurements, achieving a balance between the accuracy of predictions and the reliability of the measurements. Through this iterative process, the KF algorithm optimally refines its estimate, effectively reducing the estimation error, [15–17]. An important aspect of the KF algorithm is its ability to handle inherent model uncertainty and noisy observations. This is particularly important in the context of COVID-19 modelling, where the data is often incomplete, delayed, noisy, and subjected to reporting biases.

A variety of published studies employed the KF algorithm to address the spread of infectious diseases in epidemiological surveillance; see airborne infectious disease in [18], HIV/AIDs in [19], parameters estimation in systems biology models in [20], Ebola virus [21] and an empirical pandemic evolution in [22], the spread of measles in [23] and the relationship between deaths and air pollution [24]. The KF algorithm and its variants such as the extended Kalman filter (EKF) have been successfully utilized to model and estimate various aspects of COVID-19 dynamics. This includes the outbreak of the pandemic, parameter estimation, confirmed cases, testing data, and mobility patterns. These filters are incorporated into different epidemiological models to enhance the reliability of estimates. However, the KF algorithm was successfully applied to estimate the COVID-19 pandemic with different models and purposes. Several studies have employed the KF algorithms. For instance, in [25] utilized the EKF within a stochastic SEIRD model to predict the trend of COVID-19 spread and simultaneously estimate both model parameters and transmission state. In [26], a time-dependent SEIRD model, integrated with the EKF algorithm, was utilized to estimate the spread of COVID-19 and employed maximum likelihood estimation to assess the time-varying model parameters based on daily reported cases. An attempt was made to estimate the effective reproduction number based on daily data using the conventional KF algorithm, as documented in [27]. As detailed in [28], the EKF algorithm was integrated with the SIRD model to improve COVID-19 estimation. Moreover, the EKF algorithm was combined based on the SEIQRD model as presented in [13] to estimate the COVID-19 behaviour and the hidden states in Saudi Arabia data. Various adaptations of the KF algorithm have been employed for COVID-19 state estimation. These include the ensemble KF [29–31], unscented KF [32], extended skew KF [33], and switching KF [34]. Moreover, the importance of using the KF algorithm to estimate the hidden states or unmeasured variables in COVID-19 behaviour such as the number of susceptible, exposed, quarantined, and recovered people, given the measured data was reported in [13, 35]. Overall, the KF and its variants are proven to be a valuable tool in fighting against COVID-19 spread and helping researchers and Governments to better understand the dynamics of the disease for making more informed decisions. Motivated by existing literature, this paper introduces a novel method for conducting model selection in the context of COVID-19 spread. This approach, unique in its integration of the EKF algorithm and Bayesian inference framework, will be explained in this paper. Furthermore, this paper delves into an additional aspect of the EKF algorithm, showcasing its ability to compare pandemic models by evaluating their relative performance for Saudi Arabia’s COVID-19 data.

However, the prediction and estimation results for COVID-19 show variations among different epidemiological models which are based on the modifications of the basic SIR model framework. This concern is highlighted in [36] which potentially affects the decision-making process. Since all epidemiological models have benefits and limitations, it should be recognized where the comparisons between them could lead to a better understanding of the disease dynamics [37]. These variations are due to the model structure, parameter inference methods, data inputs and estimation techniques. In addition, some situations produce similar outcomes with different sets of parameters based on the available data, making it challenging to uniquely determine certain parameter values. This phenomenon is referred to as a non-identifiability problem as discussed in [38]. This implies that there is a lack of unification in recognising the transmission dynamics with several approximations and predictions with inconsistent results. Various methods to mitigate the impact of non-identifiability issues and to enhance the reliability of predictions are proposed. Examples include model calibration [38] and profile-likelihood methods. However, these methods may become infeasible for high-dimensional models, as reported in [39]. One possible approach to reduce the risk of non-identifiability issues is to use Bayesian inference which addresses the epistemic uncertainty by assigning a probability distribution for every given data point as demonstrated in [36]. We here demonstrate that Bayesian inference and an improved Markov chain Monte Carlo algorithm, the affine invariant ensemble Markov chain Monte Carlo algorithm, can significantly reduce the wide parameter ranges in the uniform prior and produce workable credible intervals, even in the presence of non-identifiability [40]. The likelihood pointed to the informativeness of the data where the highest likelihood lies, maybe the most likely model. In this context, to avoid these problems in future pandemic models, we have used the generic Bayesian inference method called the nested sampling algorithm [41] to make Bayesian model comparisons through the marginal likelihood or Bayesian evidence and estimate model parameters along with uncertainty quantification simultaneously. Therefore, we compare the performance of three epidemiological models. The first model is the 6D SEIQRD model proposed in [13] with time-varying parameters in different temporal segments. The second model tested in this paper is the model proposed in [33] which is an improvement over the 6D SEIQRD model in [13] by incorporating extra parameters to add more delicate dynamical characteristics in the model. The third model is the classical 4D model SIRD. A comprehensive comparison of the three models has been conducted in this paper. Then, we will explore the sensitivities of their parameters. Following this, we will introduce the 4D SIRD model and derive the reproductive number for the three pandemic models. Additionally, we will explain the role of the EKF algorithm in comparing and validating the models and enhancing the variability in the models’ estimation results.

## 2 The nonlinear pandemic models

### 2.1 The 6D SEIQRD epidemiological model

In the SEIQRD model proposed in [13], the population was divided into six compartments: Susceptible *S*(*t*), Exposed *E*(*t*), Infectious *I*(*t*), Quarantined *Q*(*t*), Recovered *R*(*t*), and Deceased *D*(*t*), as the nonlinear ordinary differential equation:

$$\begin{array}{cc} \frac{dS}{dt} & =-\beta SI+\alpha R,\\ \frac{dE}{dt} & =\beta SI-\epsilon E,\\ \frac{dI}{dt} & =\epsilon E-\gamma I-qI-dI,\\ \frac{dQ}{dt} & =qI-q_{t}Q-dQ,\\ \frac{dR}{dt} & =\gamma I+q_{t}Q-\alpha R,\\ \frac{dD}{dt} & =dI+dQ. \end{array}$$ (1)

**Fig 1:**
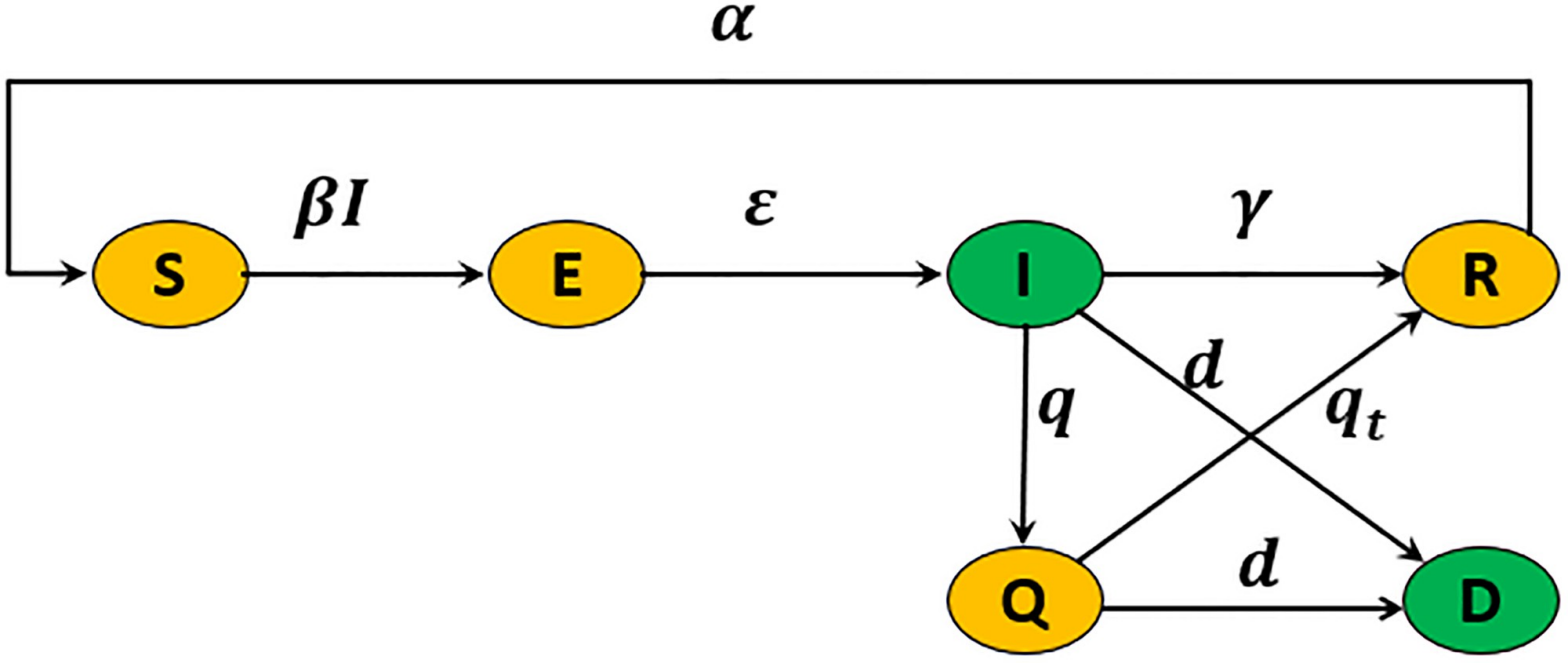
The SEIQRD model schematic diagram. The green compartments refer to the measured states in this study, while the rest are estimated.

The model parameters {*β*, *γ*, *ϵ*, *q*, *q*_*t*_, *α*, *d*} are defined as the transmission rate, recovery rate, incubation rate, quarantine rate, quarantine period, reinfection rate, and death rate respectively, where,

$$\begin{array}{c} S+E+I+Q+R+D=N, \end{array}$$ (2)

defining *N* as the total size of a population. Fig 1 shows the schematic diagram of the epidemiological system in Eq (1).

### 2.2 The 6D improved-SEIQRD epidemiological model

This section presents the improved-SEIQRD model proposed in [33] which is extended the SEIQRD model in Eq (1). The improved-SEIQRD model preserves the dimension with extra parameters to make a better representation of delicate long-term behaviour and consider the dynamic growth in the population. The extra parameters in the improved-SEIQRD model are a constant births/residents rate λ, natural death rate *μ* and the daily vaccination rate *v* of the susceptible individuals who received the vaccine per day as:

$$\begin{array}{cc} \frac{dS}{dt} & =\lambda -(\beta I+\mu +v)S+\alpha R,\\ \frac{dE}{dt} & =\beta IS-(\epsilon +\mu )E,\\ \frac{dI}{dt} & =\epsilon E-(\gamma +q+d+\mu )I,\\ \frac{dQ}{dt} & =qI-(q_{t}+d+\mu )Q,\\ \frac{dR}{dt} & =\gamma I+vS+q_{t}Q-\left(\mu +\alpha \right)R,\\ \frac{dD}{dt} & =dI+dQ. \end{array}$$ (3)

**Fig 2:**
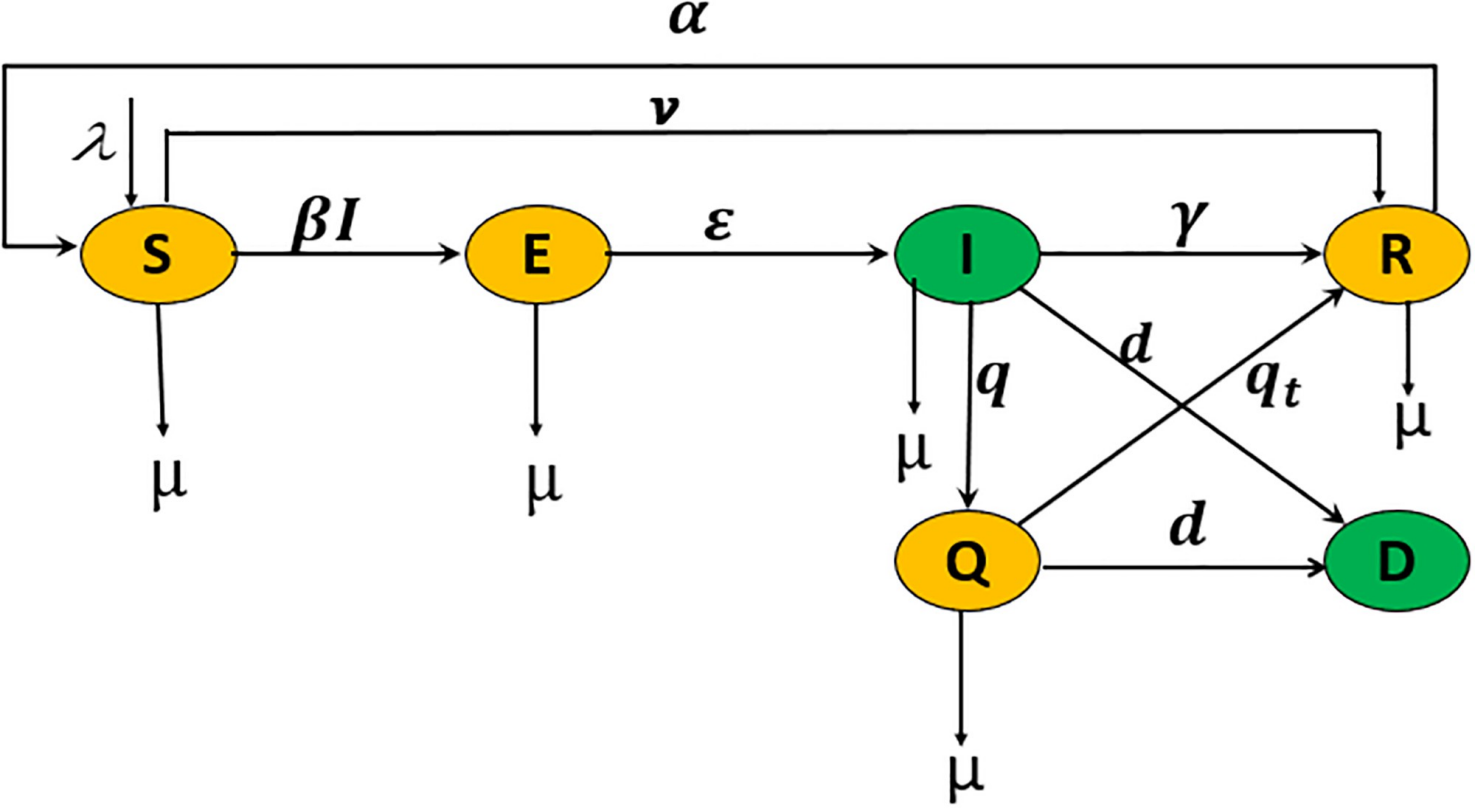
Schematic diagram of the new improved-SEIQRD model. The green compartments refer to the measured states in this study, while the rest are estimated.

The diagram of transmission dynamics of the improved-SEIQRD model is presented in Fig 2.

### 2.3 The Role of pandemic model parameters in understanding the survival mechanisms

It has been widely demonstrated that mathematical models offer a reliable methodology for supporting different nations in making optimal decisions for managing epidemics. In this section, we show a simple sensitivity analysis of some parameters and their effects on the model in Eq (1) such as the quarantine measures, incubation period, and reinfection rate. For the improved-SEIQRD model in Eq (3), we study the vaccination effect.

#### 2.3.1 Simulation results of the quarantine measures

Studying the capability of interventions such as mask-wearing, self-quarantine and social distancing to prevent a disease outbreak is essential to minimize the peak of infection, particularly in the early phase of the pandemic. The success of the quarantine measures depends on the behaviour of the population and how they follow the pandemic guidelines. We have studied the effect of quarantine measures *q* mechanisms and how they are involved in transmission. For the proposed model SEIQRD, we examined the quarantine rate *q* with three different scenarios:

1. The first scenario takes the quarantine rate if *q* = 0, where no quarantine was applied. Therefore, we will have a high rate of infection.
2. The second scenario is if 20% of the infected people were quarantined, in-home or hospitalized with *q*_*t*_ equal to 1/14 days. The infection peak will decrease to approximately half of the first scenario.
3. Finally, the third scenario concludes that if 50% of the infected people are quarantined, with *q*_*t*_ equal to 1/14 days the infection peak will sharply decrease.

**Fig 3:**
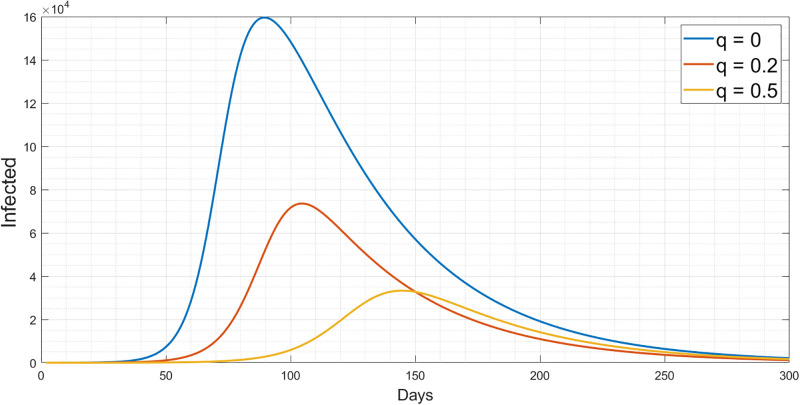
Simulation results for the different values of quarantine effect.

Subsequently, quarantine measures are vital to control the spread of the virus as shown from the modelling perspective. All these scenarios have been shown in Fig 3.

#### 2.3.2 Simulation results of the incubation period

Incubation periods can vary among individuals for different reasons, such as differences in the dynamic transmission pathways, the response to exposure to a virus, and the potential of the immune system. The duration of the incubation period for COVID-19 remains largely unknown and varies. Additionally, there is no universal agreement on the length of the incubation period for COVID-19 in the medical literature, as highlighted in [42, 43]. However, the disease is likely to be transmitted without exhibiting symptoms in exposed individuals, as they may not experience symptoms such as fever, or respiratory issues. Infections in these individuals were confirmed through RT-PCR testing on nasopharyngeal samples [44]. Additionally, approximately 30% of infected individuals remain asymptomatic, as reported in [45]. Symptoms appear between 1 to 14 days after virus exposure. Investigations into the duration of the incubation period are conducted in three different scenarios.

1. If the incubation period *ϵ* is very short, such as one day, early detection becomes more feasible, and symptoms appear shortly after. This facilitates the identification of exposed individuals, allowing for their prompt quarantine, resulting in a lower percentage of exposed individuals in the population.
2. In this scenario, symptoms develop after 14 days, corresponding to the average incubation period, ultimately reducing the percentage of exposed individuals, albeit not significantly.
3. The third scenario involves a long incubation period of 30 days, which, although rare, has been diagnosed as 24 days, as reported in [46]. This extended incubation period poses a detection challenge and increases the risk of transmission. Individuals may not exhibit any symptoms during the incubation period before developing noticeable symptoms, thereby increasing the number of exposed individuals and introducing greater uncertainty.

**Fig 4:**
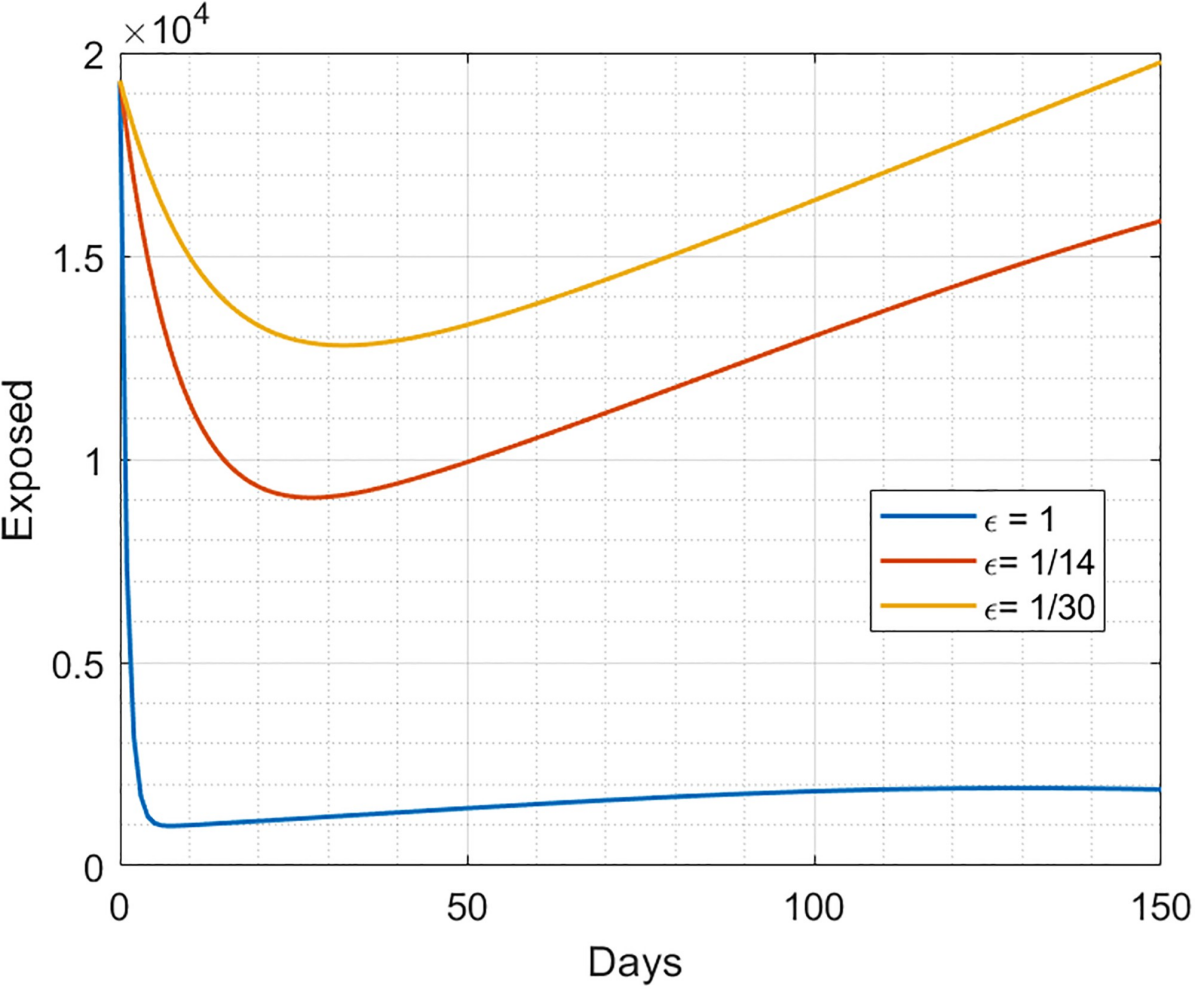
Simulation results for the different values of incubation period effect.

Fig 4 shows how the number of individuals exposed can vary depending on the different values of the incubation period *ϵ*.

#### 2.3.3 Simulation results for the reinfection rate

The actual reinfection rate with COVID-19 is difficult to determine and remains a subject of ongoing research. There is a need to understand how frequently reinfection can occur and how the emergence of new coronavirus variants might impact transmission rates [47, 48]. The reinfection may occur after 90 days as stated in [49–51]. The reinfection depends on factors such as immunity, health conditions, age, prevalence of new variants and effective vaccination. We have examined three scenarios for the reinfection:

1. The first scenario, no reinfection cases occur, i.e., *α* = 0, where individuals gain natural immunity through the initial infection. This scenario helps reduce the risk of susceptibility percentages.
2. The second scenario, reinfection occurs after 90 days, leading to a small increase in the proportion of susceptible individuals compared to the first scenario.
3. The third scenario involves reinfection occurring after a year, indicating that the disease is still ongoing and susceptibility among individuals is increasing.

**Fig 5:**
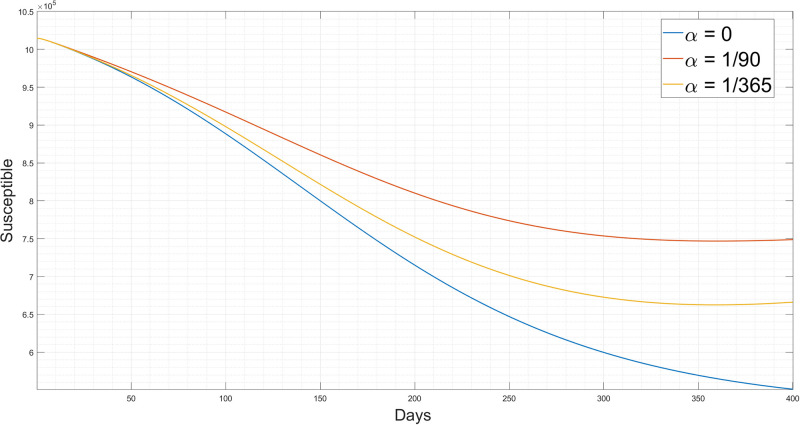
Simulation results for the different values of reinfection change for the proposed 6D SEIQRD model.

Fig 5 shows the three simulations of the reinfection rate. Furthermore, all individuals experiencing reinfection cases will return to the susceptible state, contributing to an increase in the number of susceptible cases.

#### 2.3.4 Simulation results for the vaccination rate

**Fig 6:**
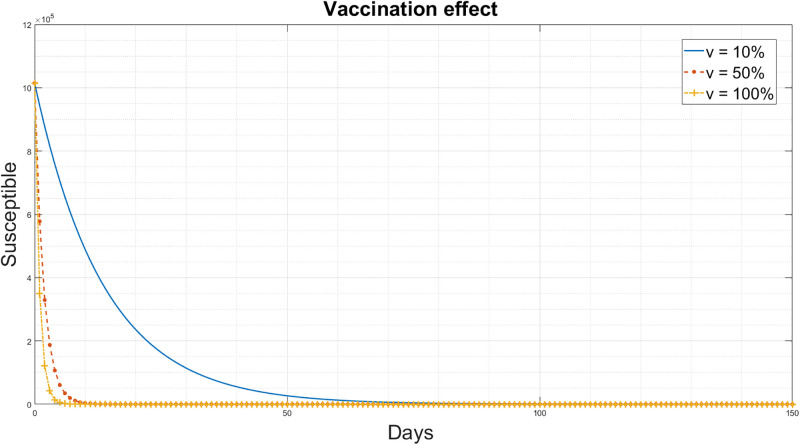
Simulation results for the different values of vaccination change for the susceptible people for the improved-SEIQRD model.

**Fig 7:**
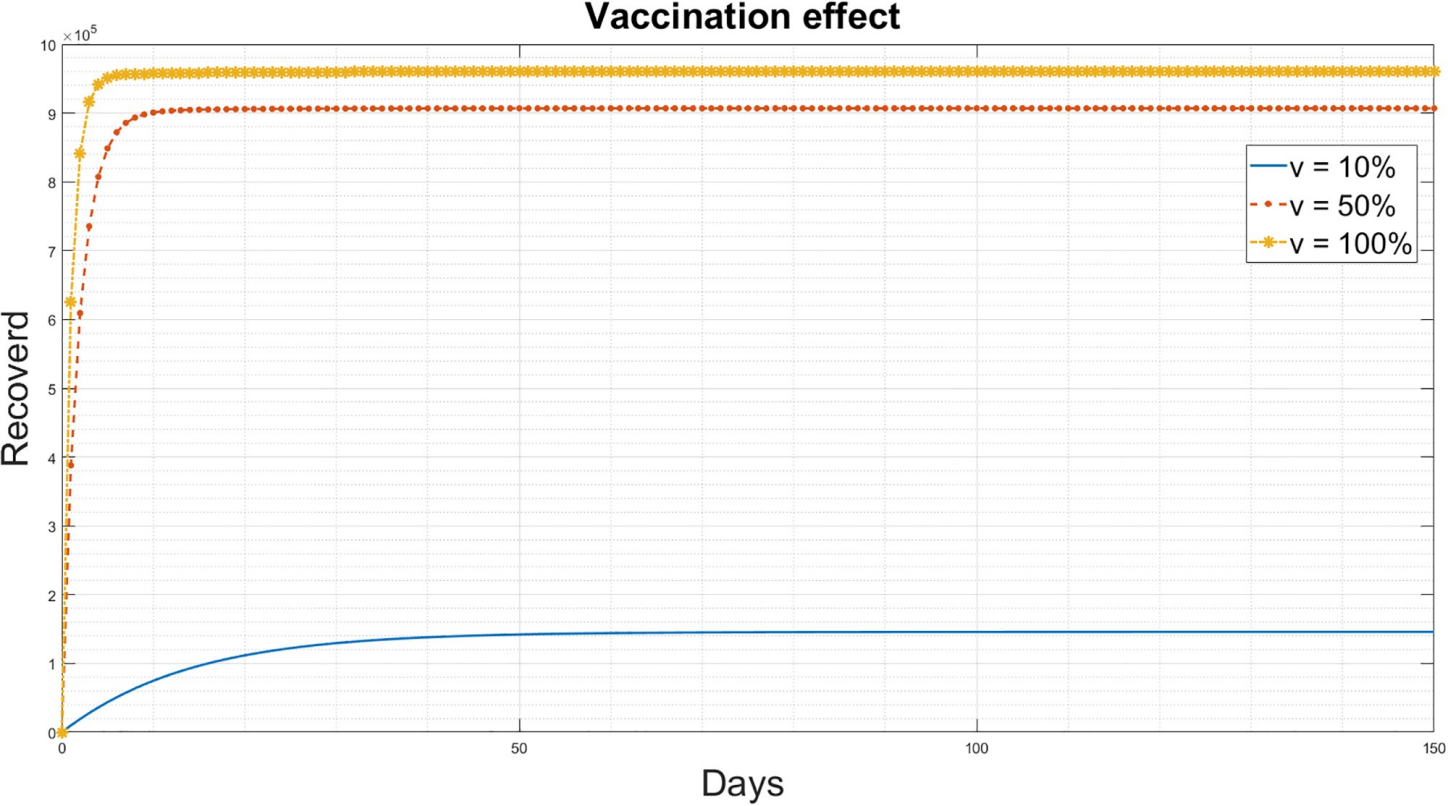
Simulation results for the different values of vaccination change for the recovered people for the improved-SEIQRD model.

The vaccination influence parameter *v* of the improved-SEIQRD model (3) was examined in two scenarios:

1. The effect of vaccination on susceptible individuals is presented in Fig 6, where vaccination potentially reduces the susceptibility of individuals to the disease. With a 100% vaccination rate, the number of susceptibles rapidly decreases and approaches zero. In contrast, if only 10% of susceptible individuals are vaccinated, the reduction in the percentage of susceptibles takes more time.
2. The vaccination effect on recovered individuals is presented in Fig 7. With a 100% vaccination rate, there is an increase in the number of recovered individuals compared to the 10% vaccination case. It can be concluded that as more people receive the vaccine, there is a corresponding rise in the number of individuals who have recovered.

### 2.4 The 4D SIRD epidemiological model

The third model we investigate in this study is the classical 4D SIRD model which is commonly used to describe the spread of infectious diseases. The SIRD model is presented with different structures in several studies such as [7, 47, 52–55]. We study the SIRD model with an additional reinfection term *α*. The SIRD model has a simple compartmental structure that divides a population into four states: Susceptible *S*(*t*), Infected *I*(*t*), Recovered *R*(*t*), and Deceased *D*(*t*), as the nonlinear differential equation:

$$\begin{array}{cc} \frac{dS}{dt} & =-\beta SI+\alpha R,\\ \frac{dI}{dt} & =\beta SI-(\gamma +d)I,\\ \frac{dR}{dt} & =\gamma I-\alpha R,\\ \frac{dD}{dt} & =dI. \end{array}$$ (4)

**Fig 8:**
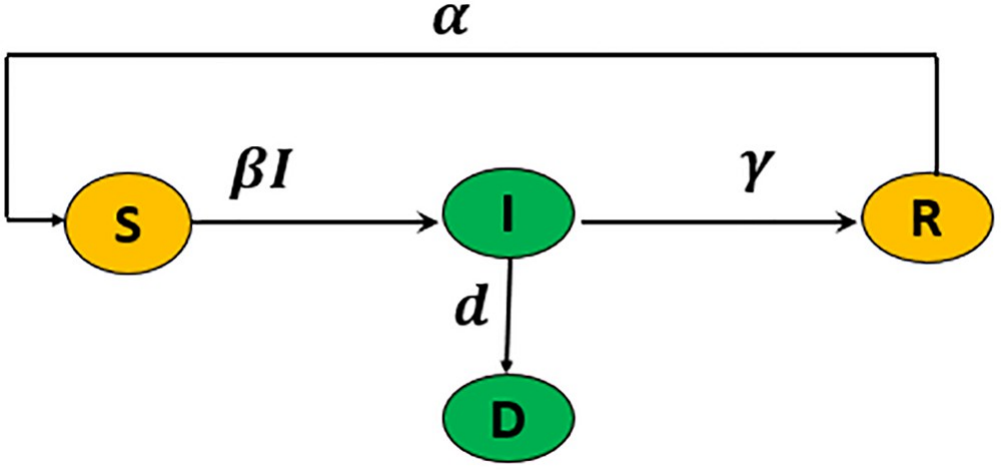
The SIRD model schematic diagram. The green compartments refer to the measured states in this study, while the rest are estimated.

Here, the model parameters are the transmission rate *β*, reinfection rate *α*, recovered rate *γ*, and death rate *d*. The total population size is *N* = *S* + *I* + *R* + *D*. The SIRD model diagram is shown in Fig 8.

The following Table 1 provides an overview of the measurable and estimated states for each model considered in the analysis, including SEIQRD, improved SEIQRD, and SIRD models.

**Table 1:** Measurable and estimated states for each class of epidemiological model.

Model	Measurable States	Estimated States
SEIQRD	Active Cases, Death Cases	Susceptible, Exposed, Quarantined, Recovered
Improved SEIQRD	Active Cases, Death Cases	Susceptible, Exposed, Quarantined, Recovered
SIRD	Active Cases, Death Cases	Susceptible, Recovered

## 3 Estimation of the time-varying reproduction number $R_{0}$

The reproduction number $R_{0}$ is the most important concept in infectious disease modelling and epidemiology for detecting the changes and severity of an outbreak in disease transmission. The $R_{0}$ is defined as the average number of secondary cases produced by a single infection in a completely susceptible population. Estimating $R_{0}$ helps public health officials, governments and researchers make informed decisions regarding disease control techniques. The $R_{0}$ is not a constant value and can fluctuate over time due to various factors, including population behaviour, vaccination campaigns, strategies adaptation and the emergence of new variants. Furthermore, the $R_{0}$ is sensitive to the model compartments and parameters estimation technique. More detailed information, about $R_{0}$ can be found in the literature e.g., [56–61]. The reproduction number $R_{0}$ can be calculated based on the next generation matrix (NGM) proposed in [60] which is a more systematic way to obtain the value of $R_{0}$ in systems involving many states to describe the evolution of the infectious disease. If $R_{0}>1$ the pandemic will spread through the population and if $R_{0}<1$ the pandemic is likely to die out naturally. Several studies have been conducted to estimate the value of $R_{0}$ based on the NGM technique for the COVID-19 pandemic, for instance, see [11, 62–64]. Here, the dynamical behaviour of the nonlinear models is different although the parameters are being estimated using the same data. Therefore, the interpretation of all system parameters across multiple models may not be identical and should only be considered in the context of a class of model.

### 3.1 Estimating the $R_{0}$ for the SEIQRD model

In this section, we will compute the $R_{0}$ for the SEIQRD model based on the next-generation matrix method (NGM) as mentioned before. To compute the $R_{0}$, we need to determine the disease-free equilibrium point $E_{0}$ for the system Eq (1), which is the state where there is no spread of the disease and at the beginning, there are no infected, exposed, quarantined, recovered and dead individuals. Then the $E_{0}$ for the model (1) is: $E_{0}=(S^{*},0,0,0,0,0)$ where S* = *N*. We will consider the subsystem exposed and infected cases of this model: *x*_*t*_ = (*E*, *I*). Then according to the NGM the model (1) can be rewritten as:

$$\begin{array}{c} {\dot{x}}_{t}=F-V, \end{array}$$ (5)

where F is the matrix corresponding to a new infection term in the model while V is the other terms of the model equations corresponding to the terms of the transition. Therefore:

$$\begin{array}{c} F=[\begin{array}{c} \beta IS\\ 0 \end{array}],V=[\begin{array}{c} \epsilon E\\ -\epsilon E+I(\gamma +q+d) \end{array}]. \end{array}$$ (6)

Evaluating the Jacobian matrices of both F and V at the disease-free equilibrium yields:

$$\begin{array}{c} DF\left(E_{0}\right)=[\begin{array}{cc} 0 & \beta S^{*}\\ 0 & 0 \end{array}],DV\left(E_{0}\right)=[\begin{array}{cc} \epsilon & 0\\ -\epsilon & \gamma +q+d \end{array}]. \end{array}$$ (7)

For simplicity, we denote $F_{1}=D(FE_{0})$ and $V_{1}=DV(E_{0})$. Then the NGM is written as:

$$\begin{array}{c} F_{1}.V_{1}^{-1}=[\begin{array}{cc} \frac{\beta S^{*}}{\left(\gamma +q+d\right)} & \frac{\beta S^{*}}{\left(\gamma +q+d\right)}\\ 0 & 0 \end{array}]. \end{array}$$ (8)

After substituting S* = *N*, the value of the $R_{0}$ which is the spectral radius *ρ* of the Eq (8) can be obtained as:

$$\begin{array}{cc} R_{0} & =\rho (F_{1}V_{1}^{-1}),\\ R_{0} & =\frac{\beta N}{\gamma +q+d}. \end{array}$$ (9)

### 3.2 Estimating the $R_{0}$ for the improved-SEIQRD model

It is important to note that each model, even if it is an extension of a previous model of the same dimension, has its own reproductive number $R_{0}$ with different representations. In this section, we will estimate $R_{0}$ for the improved-SEIQRD model since it has further parameters as explained above.

The disease free-equilibrium $E_{0}$ of the model Eq (3) by setting all the derivatives to zero is:

$$\begin{array}{cc} S_{0}^{*} & =\frac{\lambda (\alpha +\mu )}{\mu (\alpha +\mu +v)},\\ E_{0}^{*} & =0,\\ I_{0}^{*} & =0,\\ Q_{0}^{*} & =0,\\ R_{0}^{*} & =\frac{\lambda v}{\mu (\alpha +\mu +v)},\\ D_{0}^{*} & =0. \end{array}$$ (10)

Thus, the disease-free equilibrium $E_{0}$ for the improved-SEIQRD model is

$$\begin{array}{c} E_{0}=(\frac{\lambda (\alpha +\mu )}{\mu (\alpha +\mu +v)},0,0,0,\frac{\lambda v}{\mu (\alpha +\mu +v)},0). \end{array}$$ (11)

We will consider the subsystem exposed and infected cases of the model in Eq (3) with *x* = (*E*, *I*) as:

$$\begin{array}{c} \dot{x}=F-V, \end{array}$$ (12)

$$\begin{array}{c} F=[\begin{array}{c} \beta IS\\ 0 \end{array}], \end{array}$$ (13)

$$\begin{array}{c} V=[\begin{array}{c} (\epsilon +\mu )E\\ -\epsilon E+I(\gamma +q+d+\mu ) \end{array}]. \end{array}$$ (14)

Evaluating the Jacobian matrices of both F and V at the disease free equilibrium as:

$$\begin{array}{c} F_{1}=\nabla_{x}F={\left[\begin{array}{cc} 0 & \beta S_{0}^{*}\\ 0 & 0 \end{array}\right]}_{S_{0}^{*},I_{0}^{*}}, \end{array}$$ (15)

$$\begin{array}{c} V_{1}=\nabla_{x}V_{1}={\left[\begin{array}{cc} \epsilon +\mu & 0\\ -\epsilon & \gamma +q+d+\mu \end{array}\right]}_{S_{0}^{*},I_{0}^{*}}. \end{array}$$ (16)

The inverse of $V_{1}$ is

$$\begin{array}{c} V_{1}^{-1}=[\begin{array}{cc} \frac{1}{\epsilon +\mu } & 0\\ \frac{\epsilon }{\left(\epsilon +\mu )(d+\gamma +\mu +q\right)} & \frac{1}{\left(d+\gamma +q+\mu \right)} \end{array}]. \end{array}$$ (17)

Then, we calculate the

$$F_{1}V_{1}^{-1}$$

as:

$$\begin{array}{c} F_{1}V_{1}^{-1}=[\begin{array}{cc} \frac{\left(\beta \epsilon \lambda )(\alpha +\mu \right)}{\mu (\epsilon +\mu )(\alpha +\mu +v)(d+\gamma +\mu +q)} & \frac{\beta \lambda (\alpha +\mu )}{\mu (\alpha +\mu +v)(d+\gamma +\mu +q)}\\ 0 & 0 \end{array}], \end{array}$$ (18)

$$\begin{array}{cc} R_{0} & =\rho (F_{1}V_{1}^{-1}),\\ R_{0} & =\frac{\left(\beta \epsilon \lambda )(\alpha +\mu \right)}{\mu (\epsilon +\mu )(\alpha +\mu +v)(d+\gamma +\mu +q)}. \end{array}$$ (19)

### 3.3 Estimating the $R_{0}$ for the SIRD model

Estimating the reproductive number $R_{0}$ for the SIRD model in Eq (4) is relatively straightforward to derive $R_{0}$ and does not require the NGM method. The $R_{0}$ can be calculated as the ratio of the infection rate (*β*) to the rates of recovery (*γ*) and death rate (*d*), where the epidemic occurs if the number of infected individuals increases, i.e. $\frac{dI}{dt}>0$. This yields:

$$\begin{array}{cc} & \beta SI-(\gamma +d)I>0,\\ & \Rightarrow \beta SI>(\gamma +d)I,\\ & \Rightarrow \frac{\beta SI}{\gamma +d}>I,\\ & \Rightarrow R_{0}=\frac{\beta S}{\gamma +d}. \end{array}$$ (20)

Substituting the disease-free equilibrium *S* = *N* which corresponds to the situation that the whole population is susceptible to the infection at the initial stage, yielding the value of the $R_{0}$ as:

$$\begin{array}{c} R_{0}=\frac{\beta N}{\gamma +d}. \end{array}$$ (21)

## 4 Extended Kalman filter based state estimation for the 6D SEIQRD models and 4D SIRD model

The Extended Kalman filter (EKF) is an extension of the traditional KF for handling nonlinear systems [65]. The EKF algorithm operates similarly to the traditional KF algorithm, utilizing iterative processes to estimate and update system states. The EKF algorithm can estimate epidemiological models with different complexities, from simple to more complex models based on the available data. This section aims to use the EKF algorithm to compare different epidemic models’ performances and identify which one is the most accurate model with lower prediction errors. In this section, we will perform the EKF algorithm for three models, the 6D SEIQRD model in Eq (1), the 6D improved-SEIQRD in Eq (3), and the 4D SIRD model in Eq (4).

The initial step involves establishing the nonlinear state space representation for the three models. The system state vector for the SEIQRD model and improved-SEIQRD model is defined as:

$$\begin{array}{c} X_{t_{1,2}}=[\begin{array}{c} S_{t}\\ E_{t}\\ I_{t}\\ Q_{t}\\ R_{t}\\ D_{t} \end{array}]. \end{array}$$ (22)

The system state vector for the SIRD model is defined as:

$$\begin{array}{c} X_{t_{3}}=[\begin{array}{c} S_{t}\\ I_{t}\\ R_{t}\\ D_{t} \end{array}]. \end{array}$$ (23)

The system state equation in the state space model representation is described as:

$$\begin{array}{c} X_{t}=f(X_{t-1})+\xi_{t},\;\xi ∼N(0,\Xi_{t}), \end{array}$$ (24)

where *ξ*_*t*_ is the process noise which is assumed to be Gaussian with zero mean and covariance matrix Ξ, and the *f*(*x*_*t*_) represents the nonlinear differential equations for three models. For the numerical stability of the model, the number of dead individuals can be determined as *D* = (*N* − (*S* + *E* + *I* + *Q* + *R* + *D*). Now, the functions of the nonlinear system for the three models are:

$$\begin{array}{c} f_{1}\left(X\right)=[\begin{array}{c} -\beta SI+\alpha R\\ \beta SI-\epsilon E\\ \epsilon E-\gamma I-qI-dI\\ qI-q_{t}Q-dQ\\ \gamma I+q_{t}Q-\alpha R\\ d(N-(S+E+R+D) \end{array}], \end{array}$$ (25)

$$\begin{array}{c} f_{2}\left(X\right)=[\begin{array}{c} \lambda -(\beta I+\mu +v)S+\alpha R\\ \beta IS-(\epsilon +\mu )E\\ \epsilon E-(\gamma +q+d+\mu )I\\ qI-(q_{t}+d+\mu )Q\\ \gamma I+vS+q_{t}Q-\left(\mu +\alpha \right)R\\ d(N-(S+E+R+D) \end{array}], \end{array}$$ (26)

$$\begin{array}{c} f_{3}\left(X\right)=[\begin{array}{c} -\beta SI+\alpha R\\ \beta SI-(\gamma +d)I\\ \gamma I-\alpha R\\ d(N-(S+R+D) \end{array}]. \end{array}$$ (27)

Since the function *f*(*X*) describes how the populations in each compartment in the three models change based on the current state of the system.

Linearizing the nonlinear systems for the SEIQRD model using the first-order Taylor series expansion yields the Jacobian matrix as:

$$\begin{array}{c} F_{1}=(\begin{array}{cccccc} -\beta I & 0 & -\beta S & 0 & \alpha & 0\\ \beta I & -\epsilon & -\beta S & 0 & 0 & 0\\ 0 & \epsilon & \left(-\gamma -q-d\right) & 0 & 0 & 0\\ 0 & 0 & q & \left(-q_{t}-d\right) & 0 & 0\\ 0 & 0 & \gamma & q_{t} & -\alpha & 0\\ -d & -d & 0 & 0 & -d & -d \end{array}). \end{array}$$ (28)

The Jacobian matrix for the improved-SEIQRD model is

$$\begin{array}{c} F_{2}=(\begin{array}{cccccc} -(\beta I+\mu +v) & 0 & -\beta S & 0 & \alpha & 0\\ \beta I & -(\epsilon +\mu ) & \beta S & 0 & 0 & 0\\ 0 & \epsilon & -(d+\gamma +\mu +q) & 0 & 0 & 0\\ 0 & 0 & q & -(d+\mu +q_{t}) & 0 & 0\\ v & 0 & \gamma & q_{t} & -(\alpha +\mu ) & 0\\ -d & -d & 0 & 0 & -d & -d \end{array}). \end{array}$$ (29)

The Jacobian matrix for the SIRD model is

$$\begin{array}{c} F_{3}=(\begin{array}{cccc} -\beta I & -\beta S & \alpha & 0\\ \beta I & \beta S-(\gamma +d) & 0 & 0\\ 0 & \gamma & -\alpha & 0\\ -d & 0 & -d & -d \end{array}). \end{array}$$ (30)

The observation equation in the state space model representation is given as:

$$\begin{array}{c} y_{t}=H\left(X_{t}\right)+\omega_{t},\;\psi ∼N(0,\Omega_{t},), \end{array}$$ (31)

where *y*_*t*_ is the measurement vector of observed data, *H* is the observation matrix since it measures the active cases and death cases and *ω*_*t*_ is the measurement noise, assumed as a Gaussian distribution with zero mean and covariance matrix Ω. The observation matrix *H* in the case of the SEIQRD model (1) and for the improved-SEIQRD model (3) is

$$\begin{array}{c} H=[\begin{array}{cccccc} 0 & 0 & 1 & 0 & 0 & 0\\ 0 & 0 & 0 & 0 & 0 & 1 \end{array}]. \end{array}$$ (32)

The observation matrix *H* in the case of SIRD model (4) is

$$\begin{array}{c} H=[\begin{array}{cccc} 0 & 1 & 0 & 0\\ 0 & 0 & 0 & 1 \end{array}]. \end{array}$$ (33)

The recursive EKF algorithm for state estimation can be described as the following steps:

1. Start with initializing the state vector ${\hat{X}}_{0}$ and the covariance matrix *P*_0_ as:
  $$\begin{array}{cc} \hat{X_{0}} & =E[X_{0}],\\ \hat{P_{0}} & =E[\left(X_{0}-\hat{X_{0}}\right){\left(X_{0}-\hat{X_{0}}\right)}^{T}]. \end{array}$$ (34)
2. Perform the prediction of state estimates and error covariance as:
  $$\begin{array}{cc} {\hat{X}}_{t}^{-} & =f({\hat{X}}_{t}),\\ P_{t}^{-} & =FP_{t}^{+}F^{T}+\Xi_{t}. \end{array}$$ (35)
3. Perform the measurement update of the state estimate and estimation error covariance as:
  $$\begin{array}{c} {\hat{X}}_{t}^{+}={\hat{X}}_{t}^{-}+K_{t}(y_{t}-H_{t}({\hat{X}}_{t}^{-})), \end{array}$$ (36)
  $$\begin{array}{c} K_{t}=P_{t}^{-}H_{t}^{T}{\left(H_{t}P_{t}^{-}H_{t}^{T}+\Omega_{t}\right)}^{-1}, \end{array}$$ (37)
  $$\begin{array}{c} P_{t}^{+}=(I-K_{t}H_{t})P_{t}^{-}, \end{array}$$ (38)
  where *K*_*t*_ is the Kalman gain and (^−^) denotes the process before the measurements are processed which is called a priori estimate whereas ($\hat{+}$) denotes the estimate after processing the measurement and is called a posteriori estimate that presents a better estimate of the state vector *X*_*t*_.
4. Repeat the steps in 2 and 3 until all iterations are completed.

For further information on the mechanism of the EKF algorithm, refer to [66].

## 5 Methodology of Bayesian model selection

### 5.1 Bayesian model selection for the 6D SEIQRD models and the 4D SIRD model

Designing an effective model comparison strategy is an important step for distinguishing between different models and a preparedness stage against any future pandemics. Model selection in Bayesian inference within the context of epidemiology has been studied from different perspectives, with limited research available. Some existing literature on modelling comparisons of the dynamics of infectious diseases in the epidemiology field, such as the reversible jump MCMC algorithm in [67, 68], Bayes factor in [69], marginal likelihood approximated by importance MCMC in [70], and model comparisons by sequential Monte Carlo in [71, 72] are discussed. Approximate Bayesian computation (ABC) has been proposed in [73, 74] to address the problem of model choice. However, model selection is not an easy problem to tackle, and choosing an appropriate model among models remains challenging due to the added complexity of handling multiple model spaces, requiring further effort. Furthermore, selecting the most suitable model from a set of competing models to effectively characterise pathogen transmission data and disease dynamics is frequently accompanied by uncertainty [75]. This issue naturally arises in the context of COVID-19. The purpose of this paper is to show first the select the best model based on the Bayesian evidence, calculated by the nested sampling algorithm. Next, we compare the performance of the three alternative epidemiological models in terms of the predictive accuracy of the observations using the extended Kalman filters. For example, Alyami et al. [76] compared the distribution of the noise model using nested sampling by keeping the same epidemiological model. In this paper, we extend this concept by considering three different epidemiological models along with modified noise distribution in the EKF. Then in this section, we will introduce our model comparison methodology to adapt a strategy based on the evolving nature of the COVID-19 pandemic to mitigate potential risks for future scenarios. Then, we aim to make a model comparison between the SEIQRD model in Eq (1), the improved-SEIQRD model in Eq (3), and the SIRD model in Eq (4) within Bayesian inference which will be discussed in the next section.

### 5.2 Nested sampling approach for Bayesian evidence calculation

Bayesian inference estimates the posterior distribution of the unknown model parameters by combining the prior distribution with the given/observed data, represented by the likelihood function. The Bayesian inference provides a widely accepted scientific approach to model selection by comparing and assessing the marginal likelihoods or Bayesian evidence (log*Z*) of candidate models. This method identifies the best-fitted model, given the same data, similar prior range and likelihood functions [76–81]. The marginal likelihood plays an important role in parameter estimation, model comparison, and model averaging [82]. Evaluating the marginal likelihood is challenging and often being analytically intractable [83]. However, numerical methods, such as Markov chain Monte Carlo (MCMC) [84, 85], Sequential Monte Carlo [86, 87] are employed for marginal likelihood approximation. Nevertheless, Gibbs sampling, the MCMC technique, struggles in high-dimensional parameter spaces [88]. Additionally, Metropolis-Hastings MCMC faces challenges with non-Gaussian distributions and often experiences slow convergence [89]. In addressing these challenges, an alternative and new approach called the nested sampling algorithm proposed in [41, 90], is used to approximate the marginal likelihood. In this paper, we focus on the nested sampling algorithm to estimate the model parameters in time-varying and segmented time-invariant settings, which has proved to be beneficial in approximating posterior estimates and conducting model comparisons. Nested sampling is more efficient when dealing with multimodal posterior distributions, as compared to traditional MCMC methods, which may encounter convergence issues making it valuable for model comparison [91]. The advantages of nested sampling over MCMC techniques are detailed in [92]. This approach is particularly useful in the context of COVID-19 modelling, where nested sampling can effectively explore the parameter space, improve sampling accuracy, and estimate full conditional distributions. Until recently, this approach has not been adequately addressed in the context of COVID-19. Motivated by the potential advantages of the nested sampling algorithm, especially in COVID-19 modelling, this study incorporates the nested sampling algorithm based on the proposed epidemiological models to estimate the model parameters. Nested sampling allows for a thorough exploration of the parameter space, facilitating accurate sampling with minimal iterations and samples [93]. This efficiency is crucial in COVID-19 modelling, enabling faster convergence and reduced computational intensity. Moreover, the parallelization feature of nested sampling can efficiently approximate the posterior distribution along with the Bayesian evidence, which is defined for each model. The accuracy of the Bayesian evidence based on nested sampling relies on adequate sampling of the full posterior distribution, including sampling from the tails. This ensures that no regions contributing to the Bayesian evidence are overlooked [92]. This makes it a specialized method for model selection over traditional approaches. All these features of nested sampling replace the traditional MCMC sampling techniques in our study for epidemiological data analysis. We consider the nested sampling algorithm based on the multivariate Gaussian likelihood function that provides the posterior distribution to evaluate the three models which represents the average likelihood of the observed data under each model. More precisely, we evaluate the Bayesian evidence log(*Z*) for the three models by computing the likelihood of the data given the model and integrating the likelihood over the entire parameter range. In nested sampling algorithms, the evidence log(*Z*) is a quantitative measure indicating how well a model fits the observed data without being too complex which serves the Bayesian model comparison. The nested sampling was used in [81, 94] to compare different biological models. The nested sampling algorithm provides the posterior probability distribution along with the mean and variance for the quantity of interest with reliable intervals after shrinking the prior parameter space until the tolerance is achieved on the accuracy of Δ log(*Z*) < 0.001. The likelihood function is used to estimate the set of parameters {*θ*} of the three models based on the observed data denoted as L(θ). The likelihood L and the log-likelihood log(L) for the multivariate normal distribution given data *y*, mean *μ*, covariance Σ, and model parameters *θ* can be written as:

$$\begin{array}{cc} L(y;\mu ,\Sigma ,\theta ) & =\frac{1}{{\left(2\pi \right)}^{n/2}{\left|\Sigma \right|}^{1/2}}\;\text{exp}\;\{-\frac{1}{2}\prod_{i=1}^{n}{\left(y_{i}-\mu \right)}^{T}\Sigma^{-1}\left(y_{i}-\mu \right)\},\\ \text{log}\;L(y;\mu ,\Sigma ,\theta ) & =-\frac{n}{2}\text{log}\;\left(2\pi \right)-\frac{1}{2}\text{log}\;\left|\Sigma \right|-\frac{1}{2}\{\sum_{i=1}^{n}{\left(y_{i}-\mu \right)}^{T}\Sigma^{-1}\left(y_{i}-\mu \right)\}. \end{array}$$ (39)

The initialization of the nested sampling algorithm starts with several live points *N*_*live*_ which is specified according to the required resolution of the inference. It starts by drawing a set of samples or live points *N*_*live*_ from the proposed prior distribution *π*(*θ*) and then computes the likelihood of each sample L(θ) to estimate the posterior distribution for the quantity of interest. One of the main advantages of nested sampling is its ability to efficiently estimate the Bayesian evidence or the marginal likelihood of a model as well as navigating through complicated shapes of the likelihood functions with multiple modes and degeneracies. Furthermore, we used the nested sampling algorithm to estimate the entire set of the model parameters with a time-varying approach which can influence the reliability of the model selection and the credible intervals. This is a different approach as opposed to the previous study in [13] where the SEIQRD model was considered with fixed parameters. The time-dependent parameter estimation was reported in [33] for the improved-SEIQRD model. The posterior probability distribution of the model parameters is normalized by the constant called marginal likelihood or Bayesian evidence Z and can be expressed as:

$$\begin{array}{c} p\left(\theta \right)=\frac{L(\theta )\pi (\theta )}{Z}, \end{array}$$ (40)

where, *π*(*θ*) is the prior distribution, L(θ) is the likelihood function, and the term in the denominator in Eq (40) is known as the Bayesian evidence. The Bayesian evidence Z is given as the integral of the likelihood function over the prior range as:

$$\begin{array}{c} Z=\int L(\theta )\cdot \pi (\theta )d\theta . \end{array}$$ (41)

The mean posterior estimate for all the parameters E[θ|y] which is the integration of the parameter *θ* multiplied by the posterior distribution *p*(*θ*) over the entire parameter space, can be calculated as:

$$\begin{array}{c} E(\theta )=\int \theta \cdot p(\theta )d\theta . \end{array}$$ (42)

By evaluating this integral of the Eq (41) using the nested sampling approach, we can obtain an estimate of the mean posterior, which represents the expected value of the parameter given the observed data.

### 5.3 Prior selection for the Bayesian inference

We investigate the nested sampling approach with real COVID-19 data in Saudi Arabia with more extensive analysis by dividing the time into 4 successive periods according to the timeline of COVID-19 in Saudi Arabia, where each period has a different posterior probability distribution. The models will switch their initial conditions for the following period with different log(*Z*) values for each period. This approach helps us to account for the changes, jumps and fluctuations in the dynamics of the systems which may not be well captured by a single set of initial conditions and demonstrated in the result section. We used uninformative prior assigned to the uniform distribution across the parameter space for the three models. Uniform sampling is not a straightforward operation but can be constructed by incorporating the researchers’ prior experience, according to the observed data and the history of government interventions which leads to the desirable posterior distributions. The uninformative prior for the SEIQRD model is given in Table 2, for the improved-SEIQRD model in Table 3 and for the SIRD model in Table 4. It is notable from the uninformative prior tables that the prior ranges for parameters in the three models differ, attributed to the models’ structure, dimensionality and complexity of the model. Variation in prior distributions is possible for the same parameters, as shown in [95, 96]. Every model attempts to capture the target posterior aligning and being consistent with the observed data. Our investigation in Bayesian model comparison does not show the explicit specification of priors or mention of parameter values for different models, which is often lacking. For example in [97] the researchers carry out some detailed comparisons between various epidemiological models without discussing the potential prior values. The diversity in our chosen prior distributions across the three models suggests that a model’s effectiveness lies in its capacity to account for data using its credible parameter values. Conversely, using the same prior parameter values in this study is challenging, leading to less representativeness and poor performance in capturing the underlying dynamics where every model should be assigned a different weight. Moreover, we set a broad range for parameter values since the marginal likelihood, approximated by the nested sampling, can shrink the region in the parameter space to provide the posterior sample of the model parameters. Incorporating these specific prior ranges has proven to yield the best performance under the given assumptions and utility structures. While prior information might be available in certain contexts, as mentioned in clinical research, our approach utilises uninformative priors for comparative purposes, naturally leading to different posteriors. Although having a unique prior range across the models is an ideal scenario, it may not be useful in this study due to the nature of our data analysis approach. Despite the differences in prior specifications, the simulations demonstrate the models’ best fits, emphasising the effectiveness of our chosen approach.

**Table 2:** The uninformative prior distributions for the 6D SEIQRD model.

Period	*β*	*ϵ*	*γ*	*d*	*q*	*q*_*t*_	*α*
**First period**	$U\left[1.1e^{-8},5e^{-8}\right]$	U[0.001,0.31]	U[0.01,0.1]	U[0.01,0.6]	U[0,0.0001]	U[0.001,0.01]	U[0.001,0.01]
**Second period**	$U\left[1e^{-10},1e^{-9}\right]$	U[0.001,0.31]	U[0.01,0.2]	U[0.01,0.9]	U[0.001,0.05]	U[0.02,0.2]	U[0.001,0.01]
**Third period**	$U\left[1e^{-10},6e^{-6}\right]$	U[0.29,0.31]	U[0.01,0.9]	U[0.001,0.9]	U[0.001,0.01]	U[0.02,0.2]	U[0.001,0.01]
**Fourth period**	$U\left[1e^{-10},8e^{-6}\right]$	U[0.29,0.3]	U[0.1,0.9]	U[0.001,0.9]	U[0,0.001]	U[0,0.001]	U[0.0001,0.001]

**Table 3:** The uninformative prior distributions for the 6D improved-SEIQRD model.

Period	*β*	*ϵ*	*γ*	*d*	*q*	*q*_*t*_	*α*	*μ*	*v*
**First period**	$U\left[1.1e^{-10},1e^{-6}\right]$	U[0.2,0.3]	U[0.01,0.9]	$U[1e^{-6},0.9]$	U[0,0.00001]	U[0,0.00001]	U[0.00001,0.0003]	$U[2e^{-5},3e^{-5}]$	U[0,0]
**Second period**	$U\left[1e^{-10},1e^{-6}\right]$	U[0.2,0.3]	U[0.01,0.9]	$U\left[1e^{-6},0.9\right]$	U[0.01,0.9]	U[0.02,0.2]	U[0.00001,0.0003]	$U[2e^{-5},3e^{-5}]$	U[0.0001,0.001]
**Third period**	$U\left[1e^{-10},5e^{-6}\right]$	U[0.29,0.31]	U[0.001,0.9]	U[0.01,0.5]	U[0.02,0.2]	U[0.001,0.01]	U[0.00001,0.0003]	$U[2e^{-5},3e^{-5}]$	U[0.001,0.001]
**Fourth period**	$U\left[1e^{-10},8e^{-6}\right]$	U[0.29,0.3]	U[0.1,0.9]	U[0.001,0.9]	U[0,0.001]	U[0,0.001]	U[0.00001,0.0003]	$U[2e^{-5},3e^{-5}]$	U[0001,0.001]

**Table 4:** The uninformative prior distributions for the 4D SIRD model.

Period	*β*	*γ*	*d*	*α*
**First period**	$U\left[1.1e^{-10},1.2e^{-6}\right]$	U[0.01,0.9]	U[0.0001,0.5]	U[0.0001,0.003]
**Second period**	$U\left[1e^{-10},1e^{-6}\right]$	U[0.01,0.9]	U[0.01,0.2]	U[0.0001,0.003]
**Third period**	$U\left[1e^{-8},7e^{-8}\right]$	U[0.29,0.31]	U[0.01,0.9]	U[0.0001,0.003]
**Fourth period**	$U\left[1e^{-10},8e^{-6}\right]$	U[0.3,0.9]	U[0.01,0.1]	U[0.0001,0.003]

## 6 Results and discussions

### 6.1 Temporal segmentation for parameter estimation of the COVID-19 pandemic data in Saudi Arabia

Studying COVID-19 in a long-term behaviour requires a non-constant parameter estimation technique. For this reason, we adopt a time-varying approach to capture the fluctuations in the patterns. This approach has been carried out in [32] with the Kalman filter estimation showing improvements in its analysis with time-varying parameters over the constant parameters. The COVID-19 data in this study was obtained from online open-source Kaggle which has different observations of data and graphical analysis available online with daily frequency data available in [98] from 4th March 2020 to 19th October 2022. On the 2nd of March, the first case of COVID-19 was reported [99]. We divided the timeline of COVID-19 into 4 periods to have a comprehensive analysis of the different phases. Each period has a trajectory in the pandemic evolution according to changes in the outbreak, lockdown measures and vaccination campaigns. The first period is from 1 : 112 days (2nd March—21st June 2020), the second period is from 113 : 300 days (22nd July—26th December 2020), the third period is from 301 : 487 days (27th December 2020—30th June 2021), and the fourth period is from 488 : 962 days (1st July 2021—10th October 2022).

**Fig 9:**
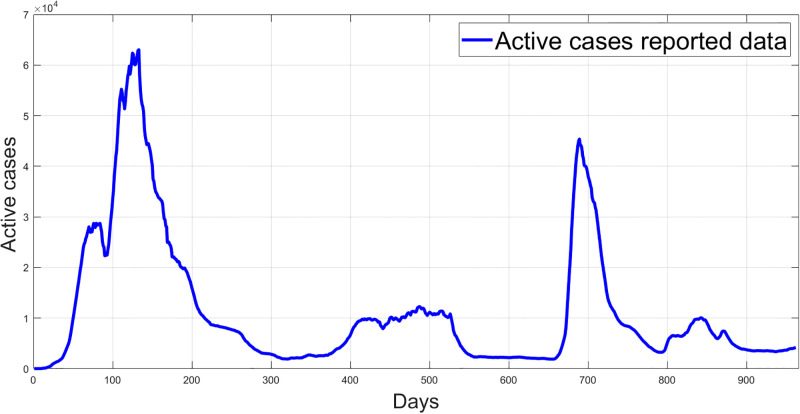
Reported active cases data in Saudi Arabia.

**Fig 10:**
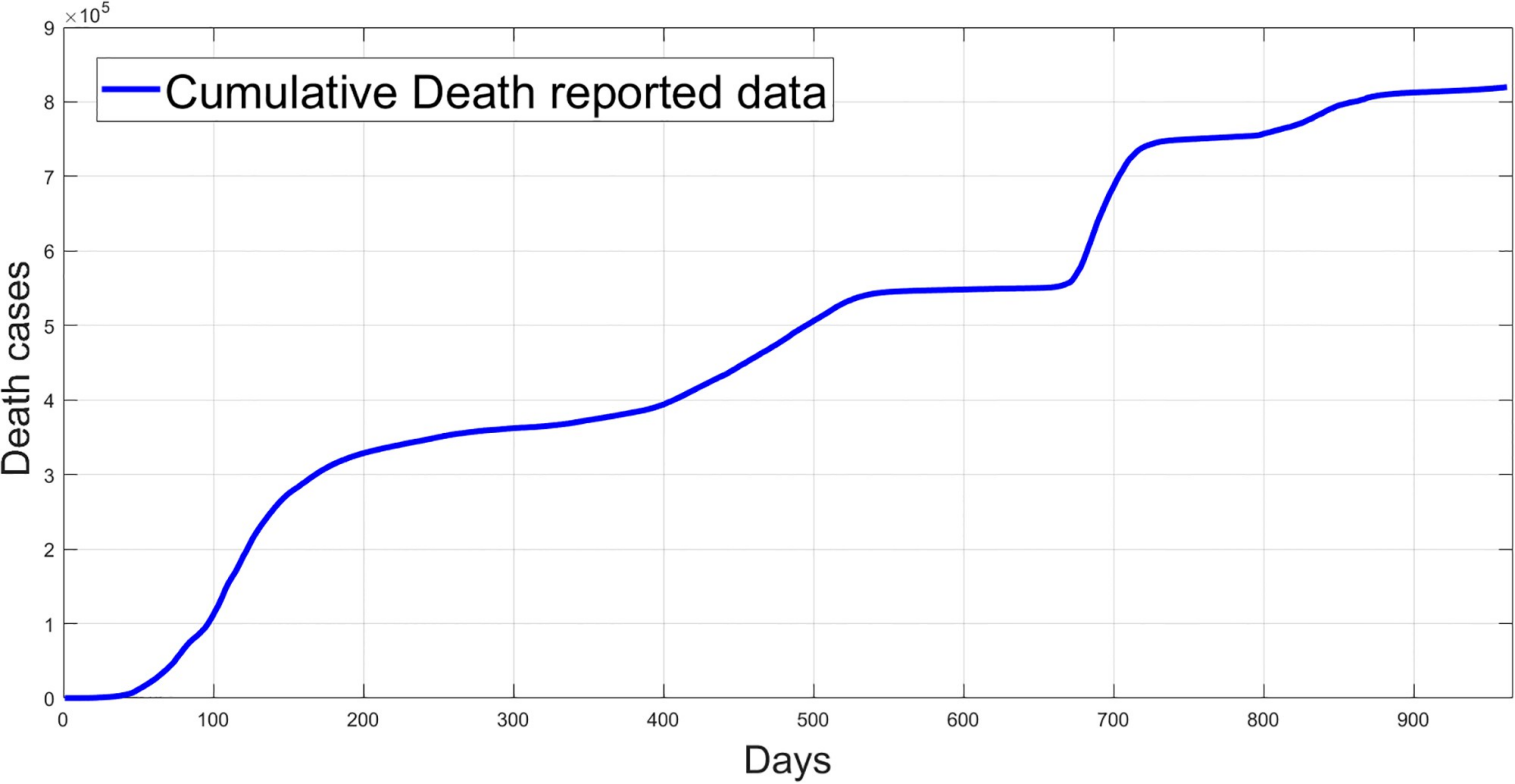
Reported cumulative death cases data in Saudi Arabia.

**Fig 11:**
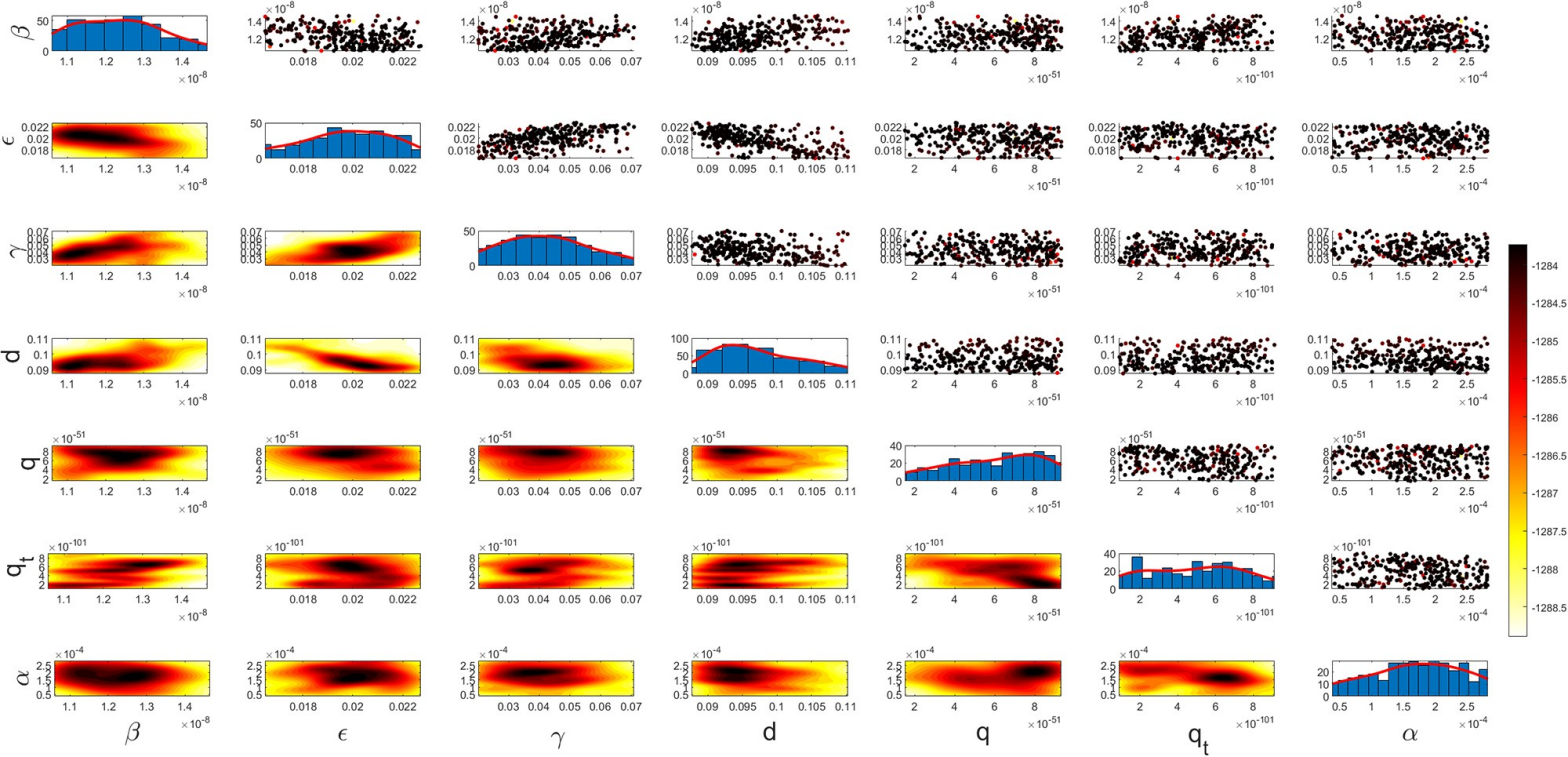
Mean posterior estimates of the SEIQRD model parameters for the first period.

**Fig 12:**
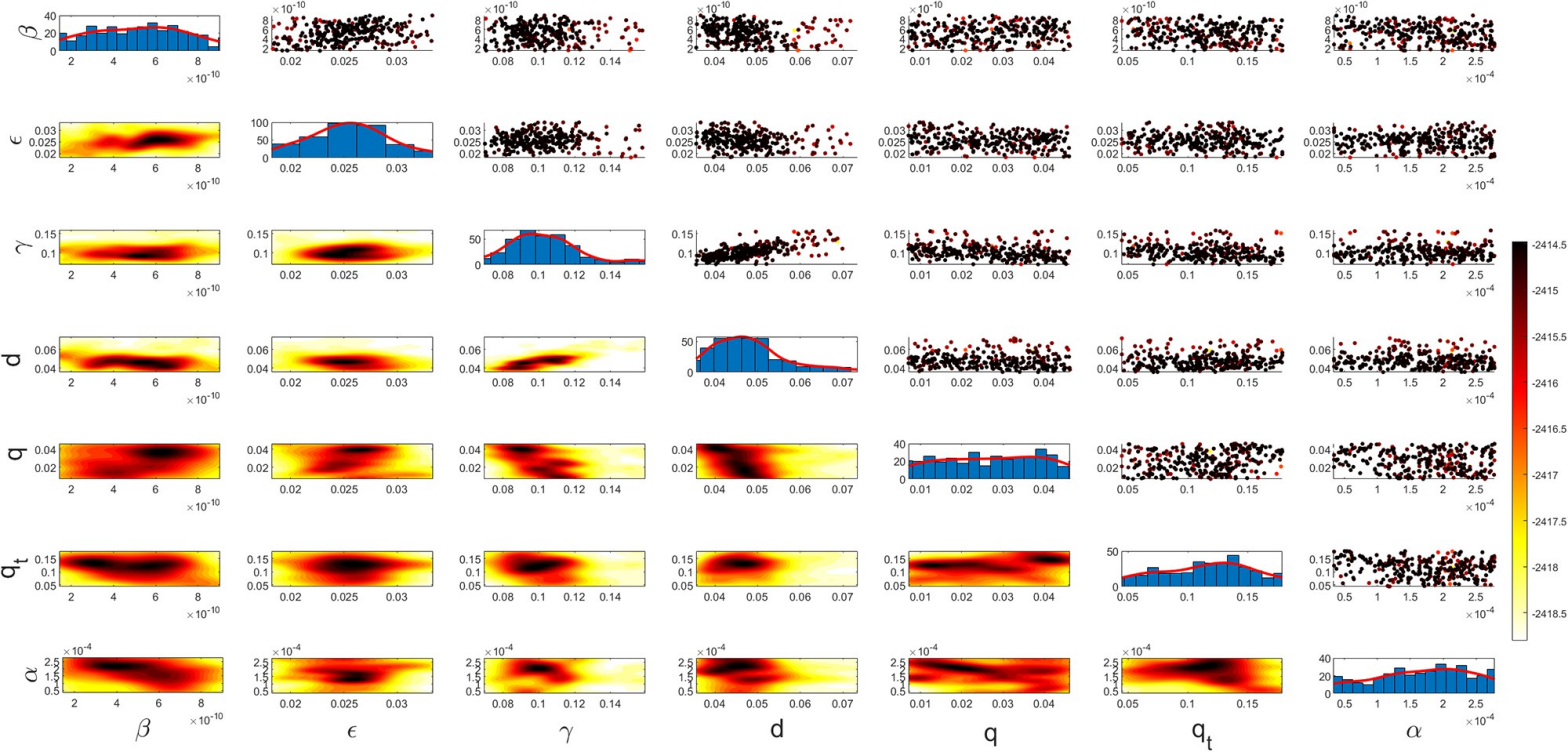
Mean posterior estimates of the SEIQRD model parameters for the second period.

**Fig 13:**
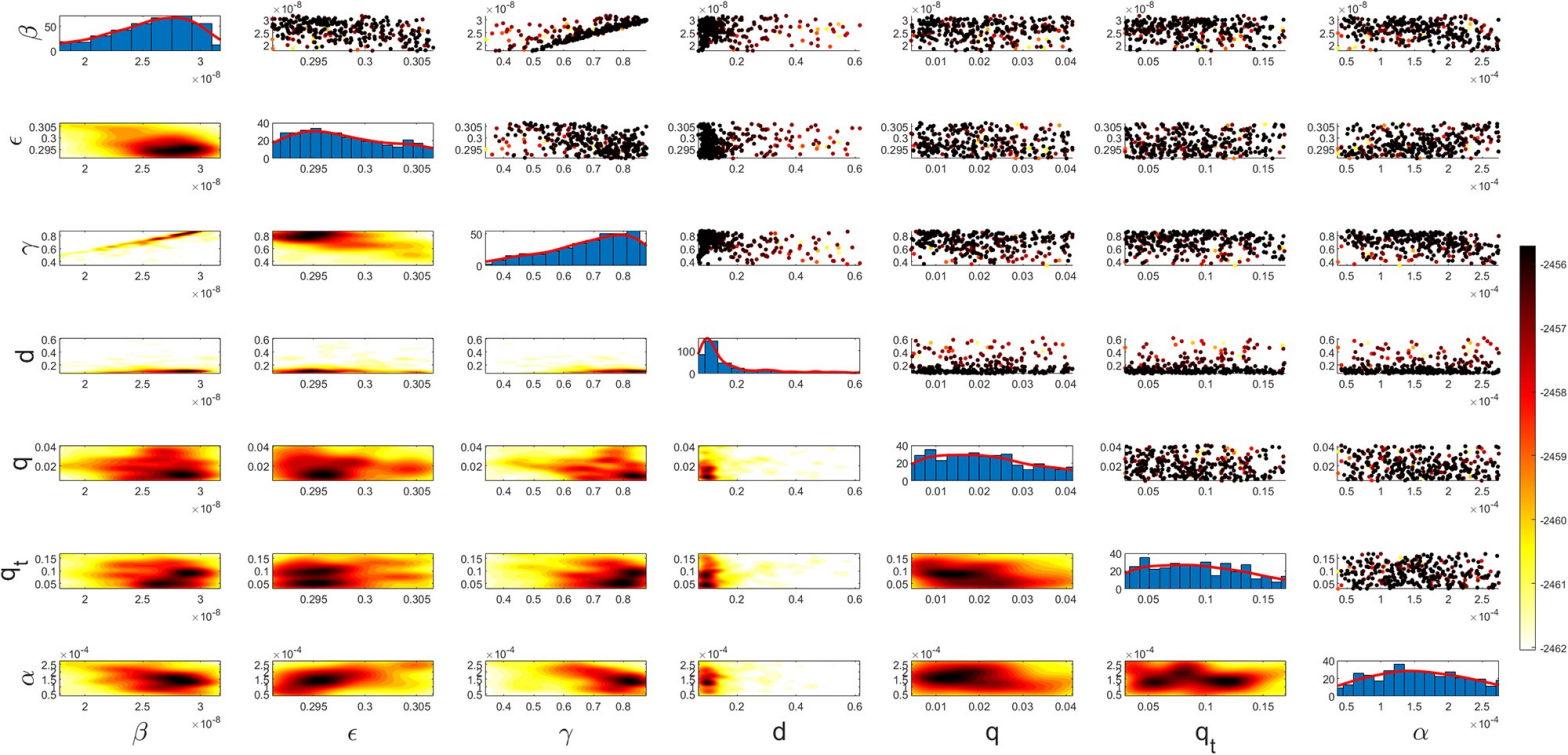
Mean posterior estimates of the SEIQRD model parameters for the third period.

**Fig 14:**
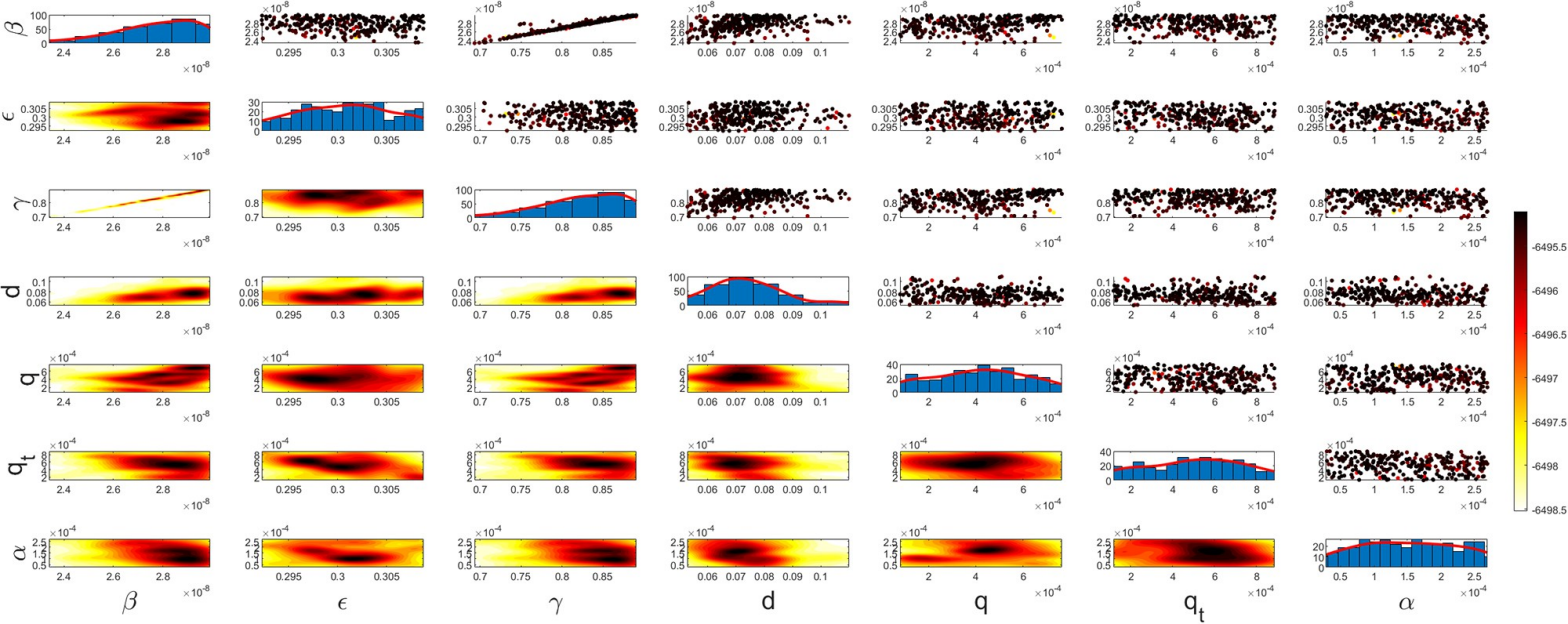
Mean posterior estimates of the SEIQRD model parameters for the fourth period.

**Fig 15:**
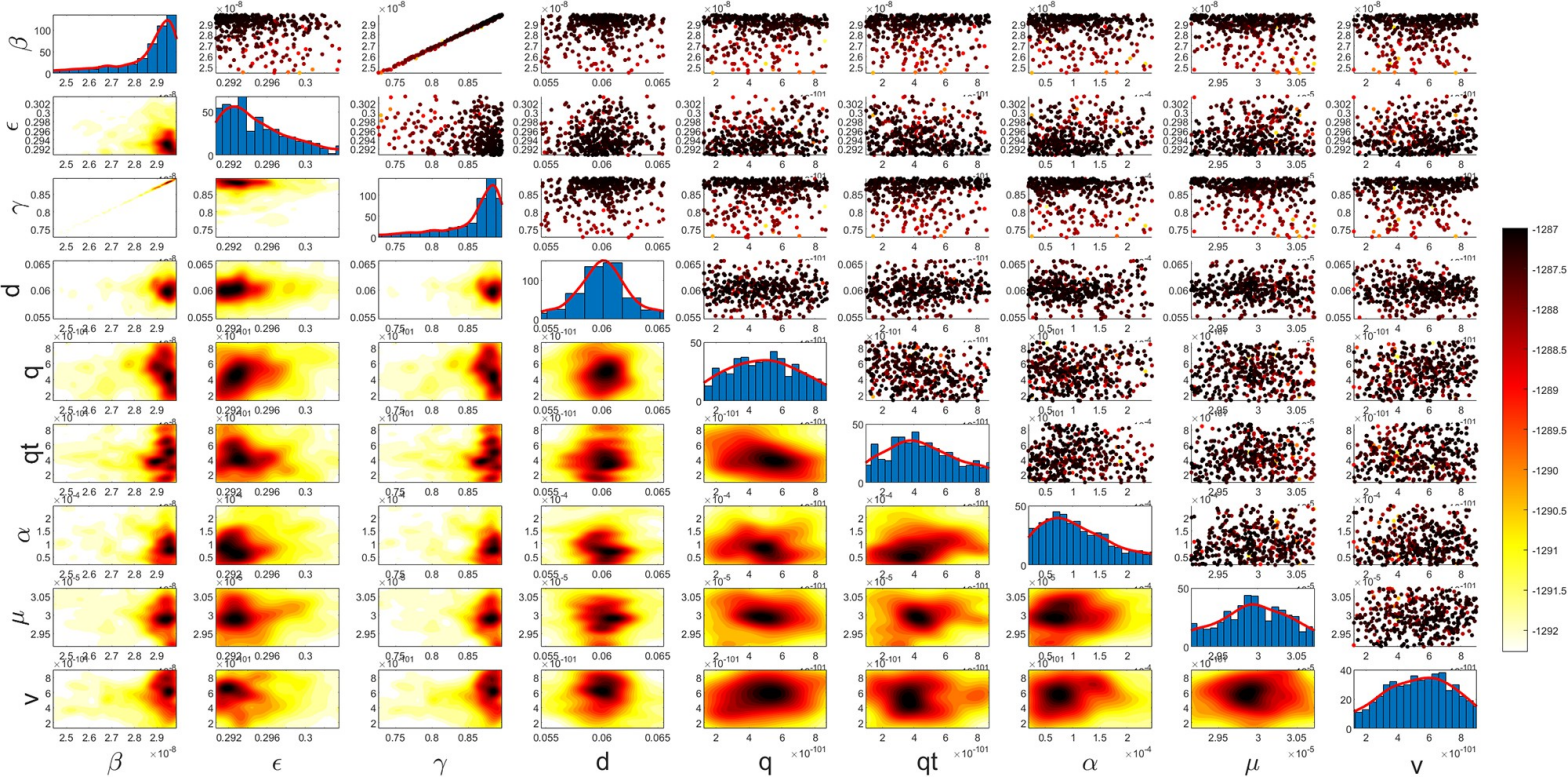
Mean posterior estimates of the improved-SEIQRD model parameters for the first period.

**Fig 16:**
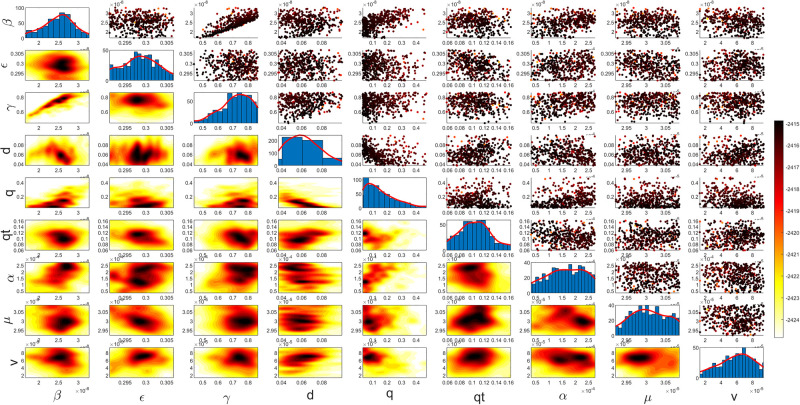
Mean posterior estimates of the improved-SEIQRD model parameters for the second period.

**Fig 17:**
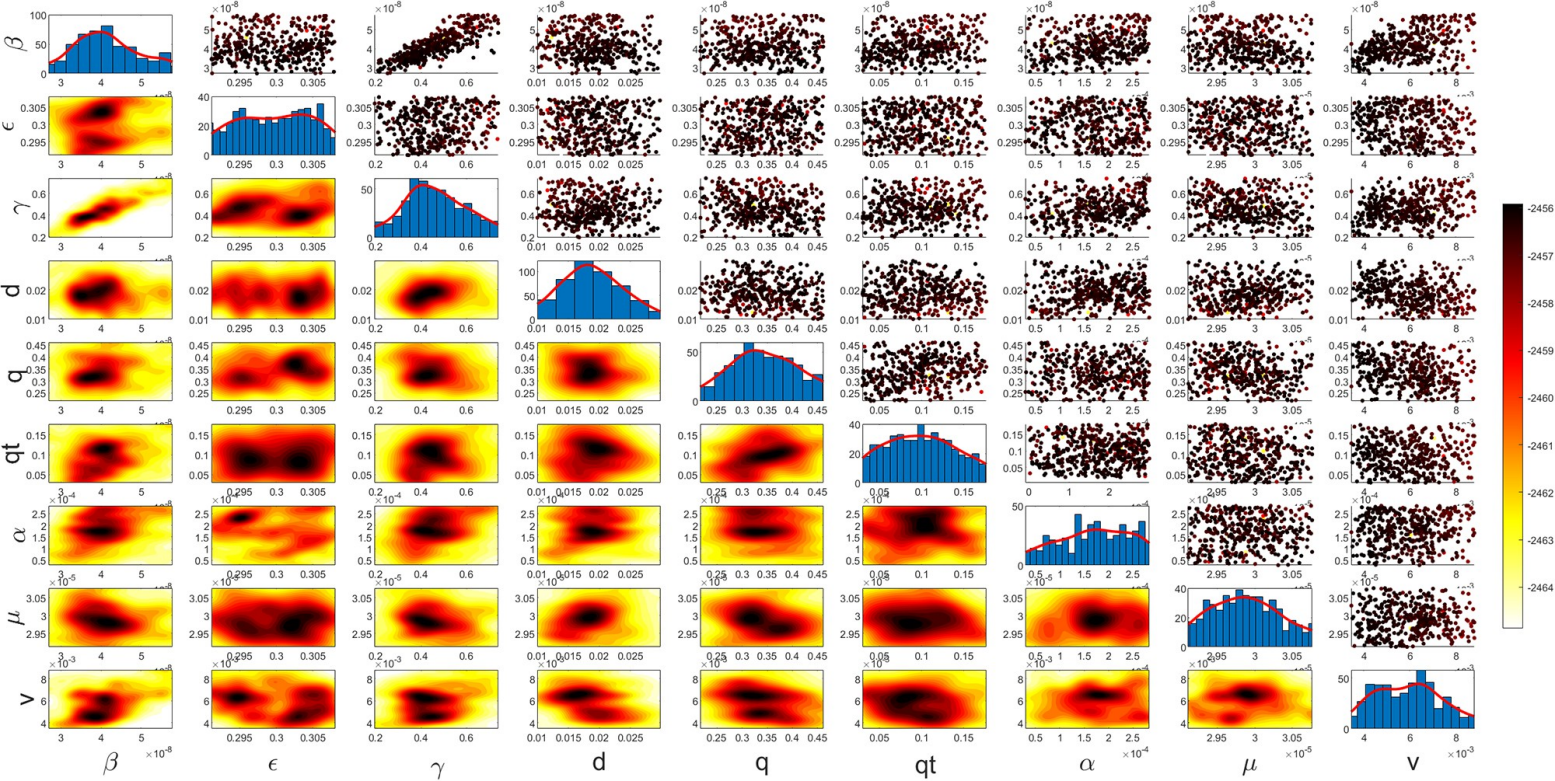
Mean posterior estimates of the improved-SEIQRD model parameters for the third period.

**Fig 18:**
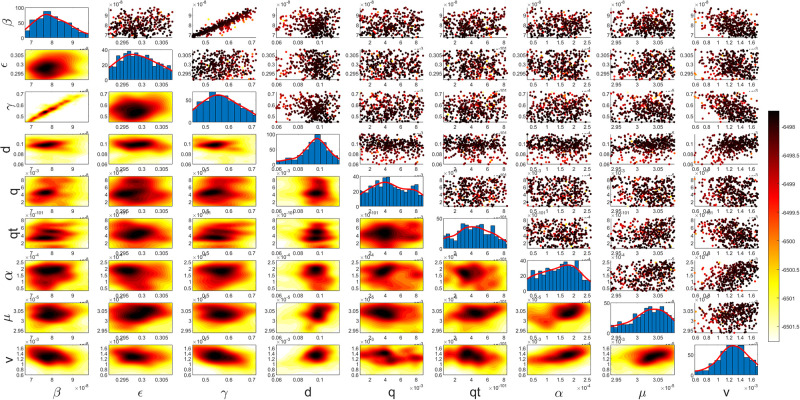
Mean posterior estimates of the improved-SEIQRD model parameters for the fourth period.

**Fig 19:**
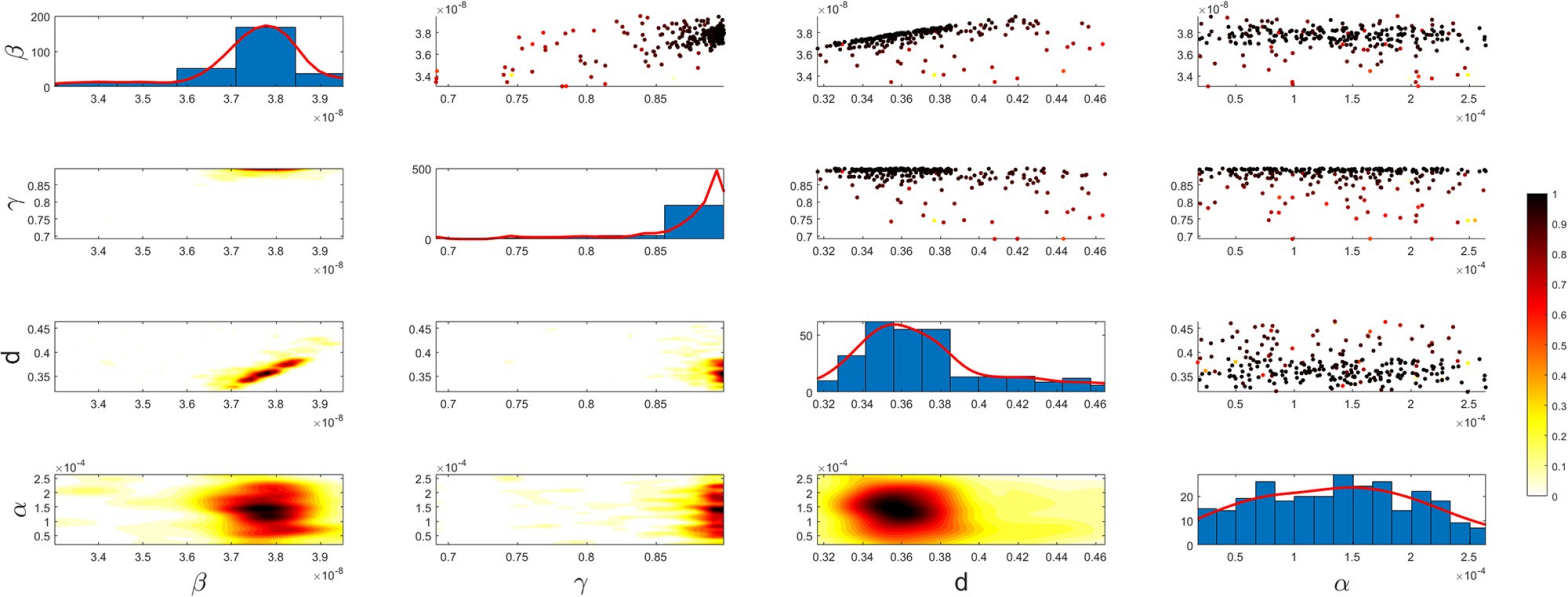
Mean posterior estimates of the SIRD model parameters for the first period.

**Fig 20:**
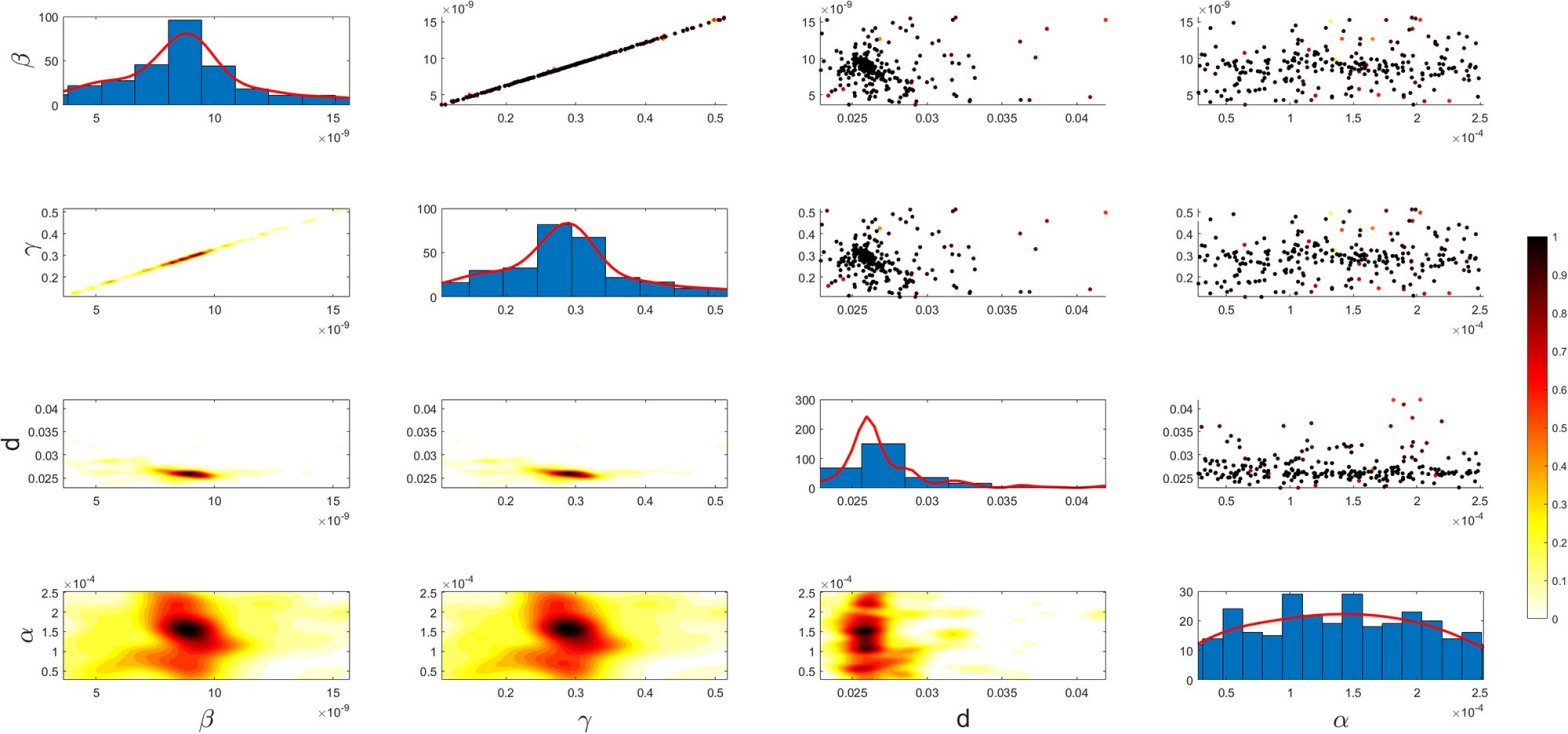
Mean posterior estimates of the SIRD model parameters for the second period.

**Fig 21:**
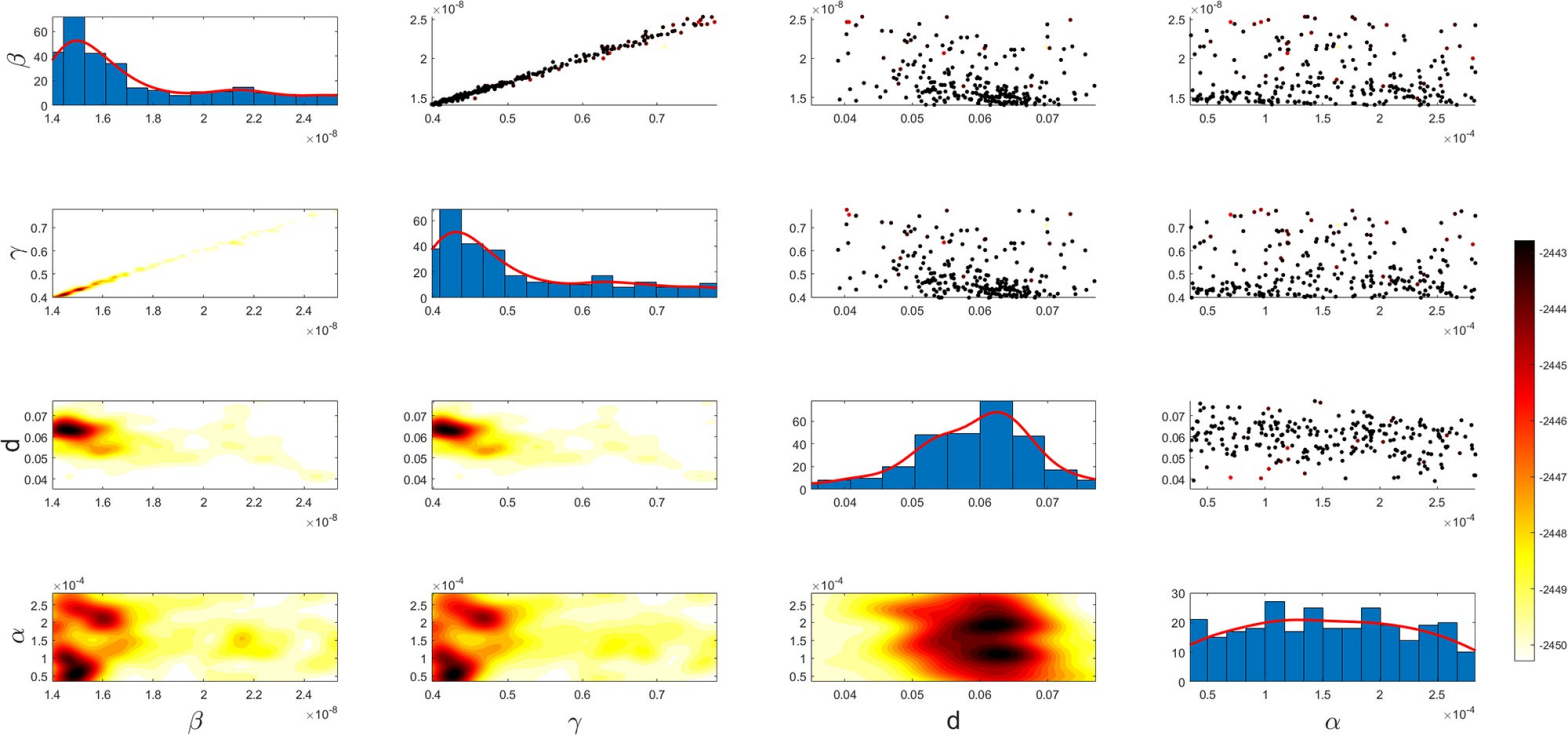
Mean posterior estimates of the SIRD model parameters for the third period.

**Fig 22:**
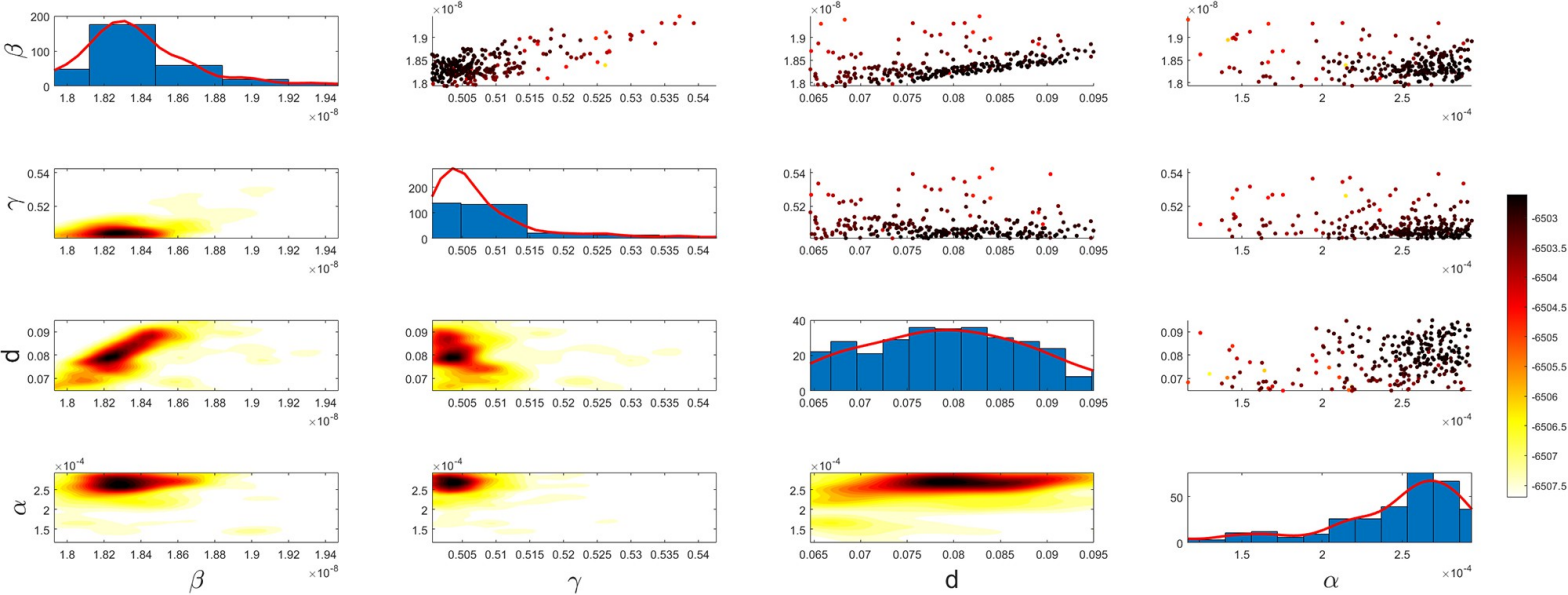
Mean posterior estimates of the SIRD model parameters for the fourth period.

Figs 9 and 10 illustrate the trend of active cases and death cases of the COVID-19 pandemic in Saudi Arabia based on the reported data respectively. They provide an overview of the fluctuating number of infected individuals and cumulative deaths while highlighting the dynamic nature of the pandemic on the Saudi Arabia population. The number of infected ≈ 66,000 people in the first period with a rapid decrease in the following periods due to the lockdown and other pandemic measures are shown in Fig 9. The decreasing number of infected cases due to the use of the smart app called (Tawakkalna) facilitates the Saudi Government to control the pandemic, report cases, update infected status, and movement permissions which is a part of Saudi Vision 2030 to digitize the services and moving forward to e-service [33, 100]. However, in the fourth period, life returns to normality and as a result, there is an increase in the infection peak on the 660th day. Fig 10 shows the cumulative number of COVID-19 death cases in Saudi Arabia highlighting the severity and impact of COVID-19 even with all the interventions and measures implemented. We implement the simulation with 962 days with the initial condition of the state vector *X*_0_ for the SEIQRD model and improved-SEIQRD model (*S*_0_ = *N* − *I*_0_ − *E*_0_, *E*_0_ = 20000, *Q*_0_ = *R*_0_ = 0), and for the SIRD model (*S*_0_ = *N* − *I*_0_, *R*_0_ = 0), where the initial numbers *I*_0_ and *D*_0_ for the three models are estimated directly from the data. At the initial time, there were no quarantined, recovered and death cases and the exposed individuals were estimated to be approximately equal to 20000. *N* is the number of Saudi Arabia’s population in this study around 35, 755, 176. Each period has different posterior estimates with different patterns and trends which are the characteristics according to the prior ranges where different policies were implemented and changed over time. Figs 11–22 show the mean posterior of the model’s parameters based on the nested sampling algorithm for the 4 periods where the principal diagonals in the figures are the 1D marginal posterior distributions of the quantity of interest. The lower triangular parts, in these Figures, show the kernel density estimate (KDE) computed by considering pairs parameters to provide the concentration of data points which may be of higher density and also lower density which is less concentrated in different regions of the plot. The upper triangular parts in the corner plots visualize the scatter-plot of the sampled log-likelihood values associated with the parameter space, determining the regions of higher and lower likelihood values where darker colours indicate higher likelihood values while the lighter-coloured regions indicate lower or worse likelihood values. Regarding the shape of the posterior distribution, it is noticeable that there is no closed-form expression for its shape. However, numerically, the nested sampling algorithm approximates the posterior distribution, and as a Gaussian approximation around the mode, we can then estimate the mean and standard deviations directly from the samples. However, it’s crucial to recognize that the exact shape of the distribution depends on both the prior specification and the data being analysed. Therefore, the posterior distribution should be viewed as a more accurate representation which is summarised in the tables using the first two moments. Additionally, regarding the non-Gaussian nature of the distribution, although the log-likelihood function follows the Gaussian distribution assumption, the posterior distribution itself may deviate from Gaussian due to the complexity of the model and data. This deviation can result in significantly higher statistical moments, which is another field of study that will be addressed in future research. Overall, the mean posterior figures effectively illustrate the patterns and trends observed in both individual and joint parameter spaces.

The mean posterior parameter estimates for the 6D SEIQRD model for the first period are shown in Fig 11, the second period in Fig 12, the third period in Fig 13 and the fourth period in Fig 14. The mean posterior parameter estimates for the 6D improved-SEIQRD model for the first period are shown in Fig 15, the second period in Fig 16, the third period in Fig 17 and the fourth period in Fig 18. The mean posterior parameter estimates for the 4D SIRD model for the first period are shown in Fig 19, the second period in Fig 20, the third period in Fig 21 and the fourth period in Fig 22. For the births/residents rate λ is assumed as a constant value with 2300 persons/day [101]. Since the entire set of model parameters are estimated by the nested sampling algorithm, from the mean posterior distributions of the three models, we obtain the best fit for them where the mean posterior estimates of the model SEIQRD are shown in Table 5, improved-SEIQRD in Table 6 and for SIRD model in Table 7. Furthermore, the posterior standard deviations or uncertainty estimates for each model parameter are presented in Table 8 for the 6D SEIQRD model, Table 9 for the 6D improved-SEIQRD model and Table 10 for the 4D SIRD model, indicating lower uncertainties for each parameter estimate and less scattered which implies that the data points around the mean posterior estimate are tightly concentrated. We initialize the nested sampling algorithm with the number of live points for the SEIQRD model as *N*_*live*_ = 70, for the improved-SEIQRD as *N*_*live*_ = 90 and *N*_*live*_ = 40 for the SIRD model which is approximately dependent on the model’s dimensionality and number of free parameters to estimate.

**Table 5:** Mean posterior estimates of the 6D SEIQRD model parameters, reproductive numbers and Bayesian evidence.

Parameter/Input	Description	1^st^period	2^nd^period	3^rd^period	4^th^period
*β*	Infection rate [Days^−1^]	1.5 × 10^−8^	5.7 × 10^−10^	2.2 × 10^−8^	2.9 × 10^−8^
*ϵ*	Incubation rate [Days^−1^]	0.0171	0.0212	0.300	0.3034
*α*	Reinfection rate [Days^−1^]	1.6 × 10^−4^	1.4 × 10^−4^	1.3 × 10^−4^	1.6 × 10^−4^
*γ*	Recovered rate [Days^−1^]	0.0492	0.1413	0.6758	0.8515
*q*	Quarantine rate [Days^−1^]	0.021	0.021	0.0269	0.00043
*q*_*t*_	Quarantine period rate [Days^−1^]	0.07	0.110	0.1013	0.000061
*d*	Death rate [Days^−1^]	0.1053	0.0389	0.1045	0.0455
*R*_0_	Reproductive number	2.0486	0.2489	1.0409	1.0963
log(*Z*)	Bayesian Evidence	-1296.1157	-2420.4774	-2476.4328	-6515.8982

**Table 6:** Mean posterior estimates of the 6D improved-SEIQRD model parameters, reproductive numbers and Bayesian evidence.

Parameter/Input	Description	1^st^period	2^nd^period	3^rd^period	4^th^period
*β*	Infection rate [Days^−1^]	2.84 × 10^−8^	1.97 × 10^−8^	4.08 × 10^−8^	6.05 × 10^−8^
*ϵ*	Incubation rate [Days^−1^]	0.296	0.301	0.298	0.301
*α*	Reinfection rate [Days^−1^]	1.81 × 10^−4^	1.32 × 10^−4^	1.53 × 10^−4^	1.85 × 10^−4^
*γ*	Recovered rate [Days^−1^]	0.852	0.495	0.483	0.584
*q*	Quarantine rate [Days^−1^]	0.02	0.023	0.027	0.005
*q*_*t*_	Quarantine period rate [Days^−1^]	0.07	0.14	0.11	0
*μ*	Natural death rate [Days^−1^]	3.01 × 10^−5^	3 × 10^−5^	3 × 10^−5^	2.99 × 10^−5^
*v*	Vaccination rate [Days^−1^]	0	0.002	0.005	7.25 × 10^−4^
*d*	Death rate [Days^−1^]	0.061	0.066	0.067	0.057
*R*_0_	Reproductive number	2.39	1.91	0.16	1.64
log(*Z*)	Bayesian Evidence	-1304.4222	-2427.1424	-2472.2386	-6519.6187

**Table 7:** Mean posterior estimates of the 4D SIRD model parameters, reproductive numbers and Bayesian evidence.

Parameter/Input	Description	1^st^period	2^nd^period	3^rd^period	4^th^period
*β*	Infection rate [Days^−1^]	3.7 × 10^−8^	9.8 × 10^−9^	1.8 × 10^−8^	1.8 × 10^−8^
*α*	Reinfection rate [Days^−1^]	1.5 × 10^−4^	1.7 × 10^−4^	1.4 × 10^−4^	2.4 × 10^−4^
*γ*	Recovered rate [Days^−1^]	0.852	0.495	0.483	0.584
*d*	Death rate [Days^−1^]	0.3660	0.0279	0.0651	0.0806
*R*_0_	Reproductive number	1.0825	0.9866	1.0237	1.0216
log(*Z*)	Bayesian Evidence	-1342.4969	-2443.0191	-2447.9587	-6514.8361

**Table 8:** Posterior standard deviation estimates of the 6D SEIQRD model parameters.

Parameter	1^st^period	2^nd^period	3^rd^period	4^th^period
*β*	4.72. × 10^−10^	1.73 × 10^−10^	7.67 × 10^−9^	4.14 × 10^−9^
*ϵ*	0.0100	0.0274	0.0051	0.0049
*α*	7.662 × 10^−8^	6.99 × 10^−8^	7.28 × 10^−8^	7.48 × 10^−8^
*γ*	0.0149	0.0090	0.2289	0.1295
*q*	0.0003	0.0106	0.0137	0.0002
*q*_*t*_	0.0022	0.0476	0.0445	0.0002
*d*	0.0034	0.0394	0.0686	0.0154

**Table 9:** Posterior standard deviation estimates of the 6D improved-SEIQRD model parameters.

Parameter	1^st^period	2^nd^period	3^rd^period	4^th^period
*β*	2.676 × 10^−10^	8.354 × 10^−11^	8.87 × 10^−11^	8.77 × 10^−11^
*ϵ*	0.0050	0.0053	0.0049	0.0049
*α*	7.52 × 10^−9^	6.70 × 10^−9^	6.49 × 10^−9^	7.568 × 10^−9^
*γ*	0.0834	0.1960	0.1708	0.1183
*q*	0.0001	0.1757	0.1115	0.0025
*q*_*t*_	0.0000	0.0403	0.0427	0
*μ*	4.33 × 10^−10^	4.71 × 10^−10^	4.82 × 10^−10^	4.40 × 10^−10^
*v*	0.0000	0.0000	0.0013	0.0003
*d*	0.061	0.066	0.067	0.057

**Table 10:** Posterior standard deviation estimates of the 4D SIRD model parameters.

Parameter	1^st^period	2^nd^period	3^rd^period	4^th^period
*β*	3.44 × 10^9^	3.74 × 10^−9^	3.92 × 10^−9^	8.92 × 10^−10^
*α*	7.49 × 10^−9^	7.544 × 10^−9^	8.45 × 10^−8^	7.17 × 10^−9^
*γ*	0.1351	0.1263	0.1326	0.0271
*d*	0.0475	0.0052	0.0119	0.0097

Additionally, the reproduction number $R_{0}$ is estimated for each period to quantify the effectiveness of the intervention strategies and assess the outbreak of the virus. The critical values of $R_{0}>1$ lie on the first period for all three models. It is notable that the variation in $R_{0}$ between the models is significant although we used the same modelling techniques where these differences indicate uncertainty surrounding the modelling of COVID-19. The lowest value of $R_{0}$ lies in the third period of the improved-SEIQRD model in Table 6 which reflects the effect of vaccination, leading to significant improvements in reducing the value of $R_{0}$. It is consistent with a large number of people being vaccinated in this period where 17 million doses of COVID-19 vaccination were conducted as mentioned in [102] which reduces the severity of the disease. The improved-SEIQRD model takes into account the vaccination in its parameters contrary to the SEIQRD model and the SIRD model. There is an increase in the value of the $R_{0}$ in the fourth period and there may be potential reasons behind the different factors, such as the effectiveness of the vaccine, number of vaccination days, vaccine distribution and herd immunity. However, the total size of the $R_{0}$ for the whole period ranges between (4-6) for all three models which remains consistent. Moreover, we evaluate the Bayesian evidence for the three models by computing the likelihood of the data given by the model and integrating over the parameter space. From Table 5 for the SEIQRD model the average value of log(*Z*) = −3177.230; from Table 6 for the improved-SEIQRD model the average value of log(*Z*) = −3180.1055 and for the SIRD model the average value of log(*Z*) = −3187.5777. The number of likelihood calls (*N*_*like*_), evaluated for each model also changes depending on the dimension of searchable parameters. For the SEIQRD model: *N*_*like*_ = 1931, for the improved-SEIQRD model: *N*_*like*_ = 3003 and for the SIRD model: *N*_*like*_ = 699, indicating the overall computational burden for posterior inference and evidence calculation. The SEIQRD model has a higher log(*Z*) value, indicating a better match to explain the observed data as compared to the improved-SEIQRD model and the SIRD model with a lower log(*Z*) value. However further analysis is needed to assess the root mean square error (RMSE) based on the EKF using these nonlinear ODE models which will be discussed in the following sections.

### 6.2 The EKF algorithm tuning in the SEIQRD model, improved-SEIQRD model and SIRD model

The initial state vectors *X*_0_ for the three models were chosen appropriately as we initialize the models in the above section. The initial covariance matrix *P*_0_ for both model 6D is (diag[10, 15, 1, 2, 1, 1]) and for the 4D model is chosen as (diag[15, 10, 12, 1]). The initial covariance matrix *P*_0_ defines the uncertainty or variance associated with the initial state estimates. These parameters are determined through a trial-and-error approach which gives the best fit. The system covariance matrix Ξ for both 6D estimated as Ξ = 500 × *I*_6×6_ and for 4D the Ξ = 500 × *I*_4×4_. The system covariance matrix Ξ generally can not be determined exactly and intuitively estimated due to being related to the noise in the system which becomes more difficult in practice to estimate it, particularly with high dimensional models. The appropriate covariance matrix Ξ covers how much uncertainty in the physical systems can be tolerated while operating the Kalman filter with optima]l performance and we found the chosen value fits the three models.

### 6.3 The effect of changing the measurement covariance matrix in the EKF algorithm

The measurement covariance matrix Ω is related to the measured data and in this study, we have measured the active cases *I*, and death cases *D*. We test two matrices where the first one is arbitrarily chosen by trial and error tuning Ω = diag[100, 1000] and the second measurement covariance matrix follows a systematic design introduced in [76] by studying the discrepancy between the processed data and the model predictions. Since these residuals provide valuable information about the uncertainties in the model. Then by quantifying these uncertainties, we update the noise covariance estimation which enhances the accuracy of the state estimates. Then for the second case we chose, Ω = diag[82, 989.2]. We used the same covariances for all three models to guarantee a fair comparison of the estimated states within the EKF algorithm. The comparison between these two values of the Ω is presented in Table 11.

**Table 11:** Comparison of the RMSE values for different EKF models with two measurements covariance matrix Ω.

EKF	Ω = diag[100, 1000]		Ω = diag[82, 989.2]	
	Infected Error	Death Error	Infected Error	Death Error
EKF-SEIQRD	9.1899	32.4296	**7.843**	**32.1418**
EKF-improved-SEIQRD	10.492	36.8938	**8.9272**	**36.6875**
EKF-SIRD	3.9226	60.5832	**3.4571**	**60.1815**

### 6.4 The EKF algorithm performance for the SEIQRD model, improved-SEIQRD model and SIRD model

**Fig 23:**
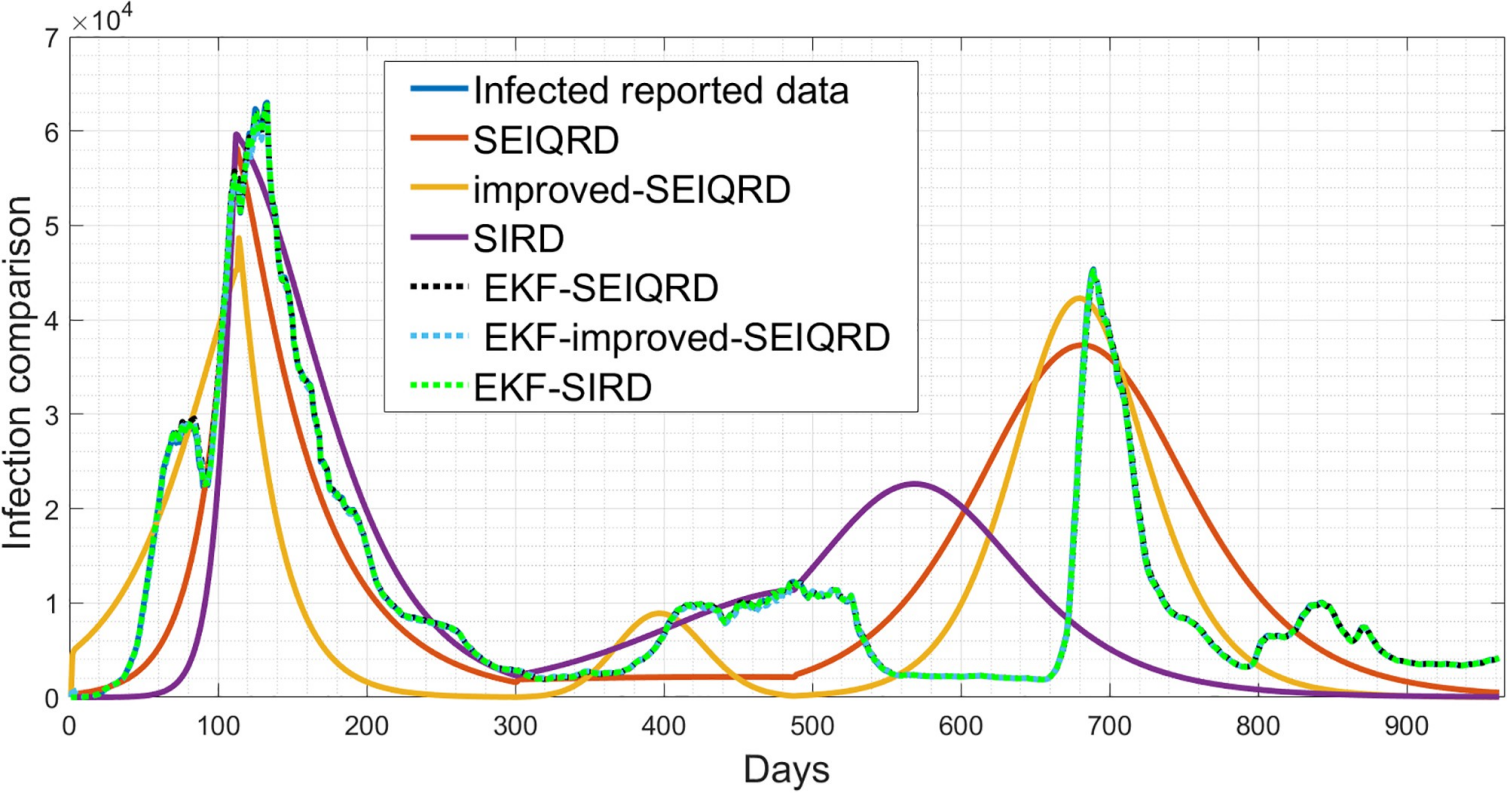
Comparison of the prediction of reported data using the SIRD, SEIQRD, SIRD-EKF and SEIQRD-EKF for active cases.

**Fig 24:**
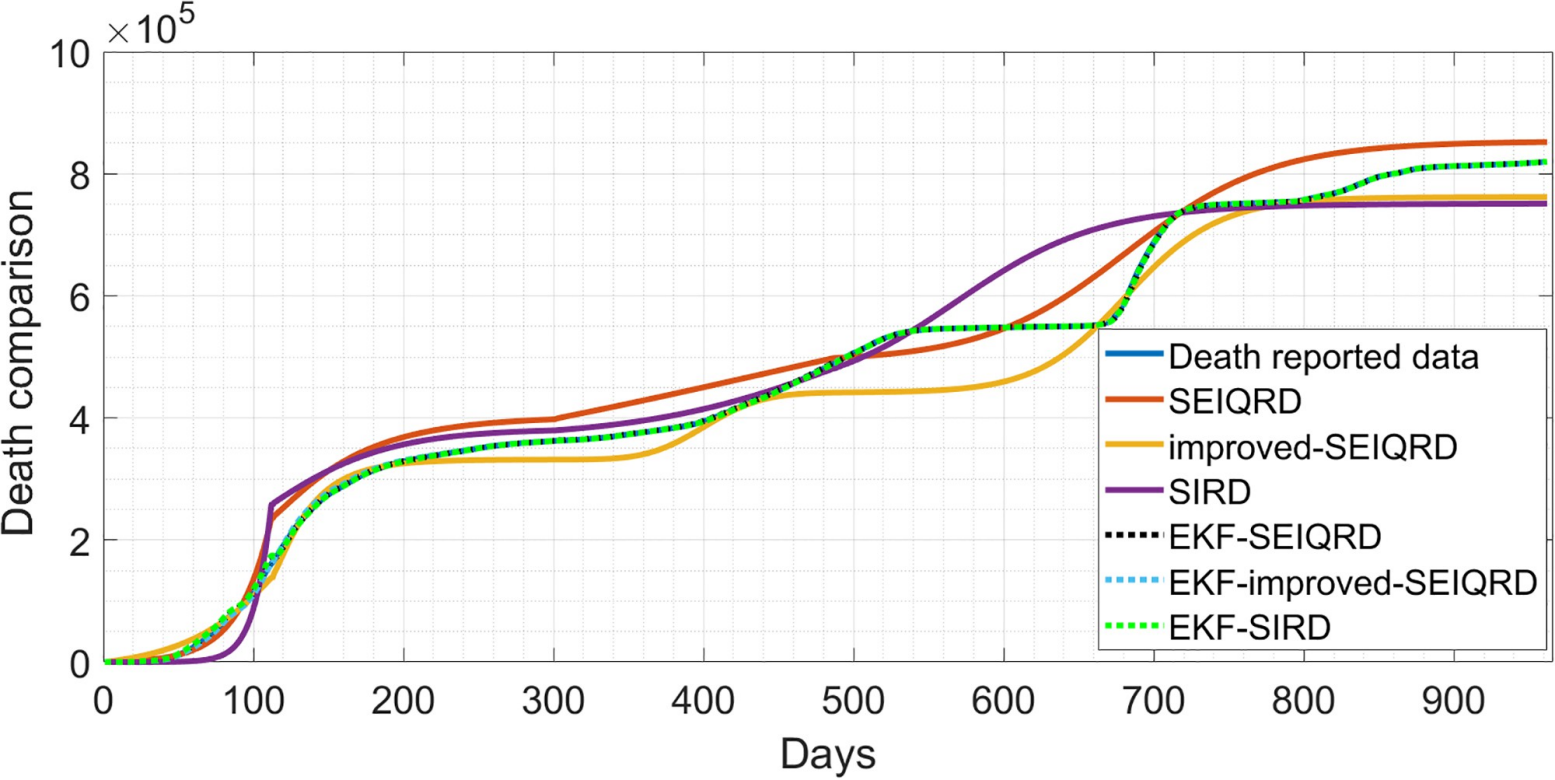
Comparison of the prediction of reported data using the SIRD, SEIQRD, SIRD-EKF and SEIQRD-EKF for cumulative death cases.

**Fig 25:**
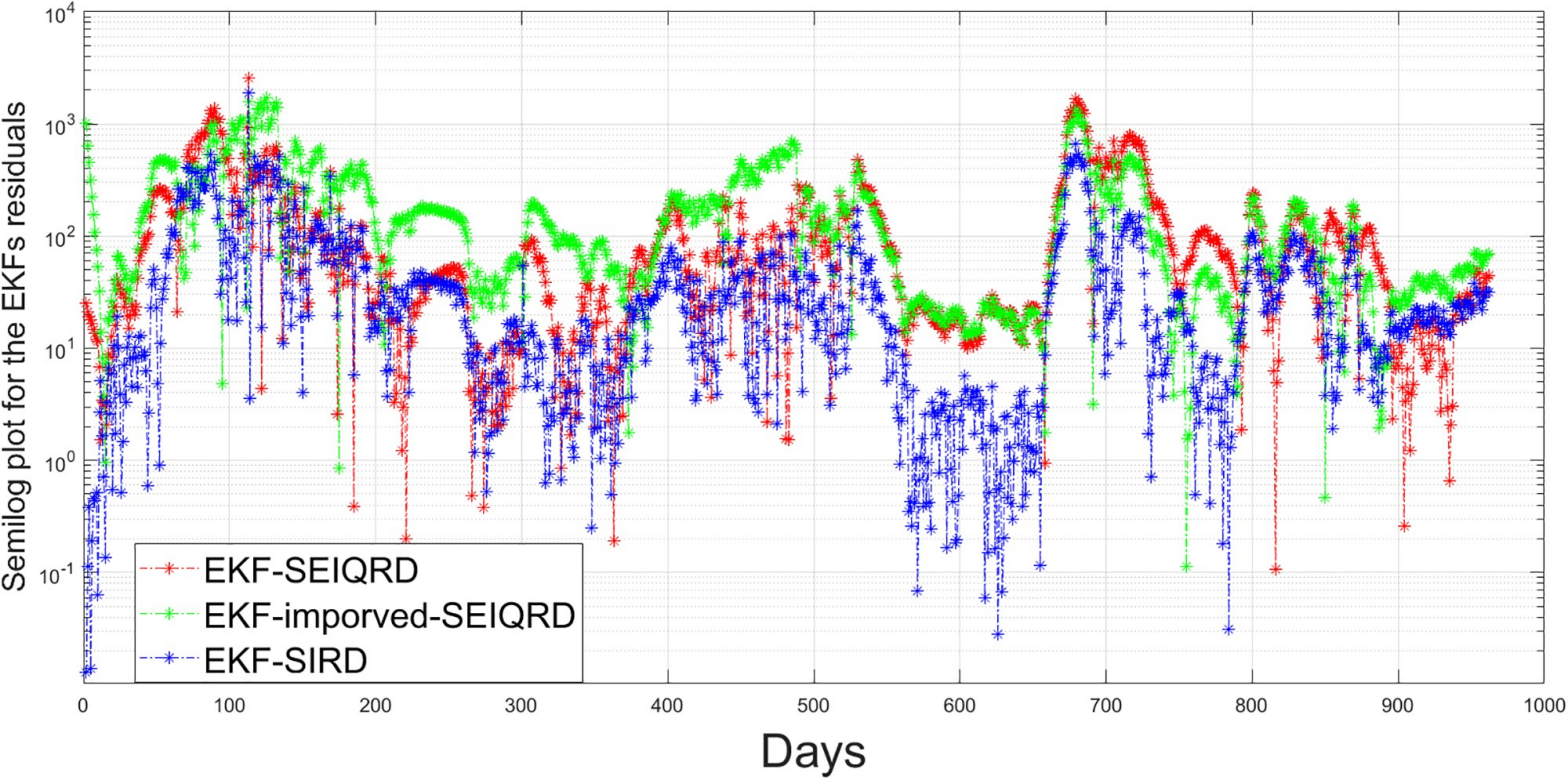
Comparison of residuals estimations for active cases: Assessing the EKF-SEIQRD, EKF-improved-SEIQRD, and EKF-SIRD models.

**Fig 26:**
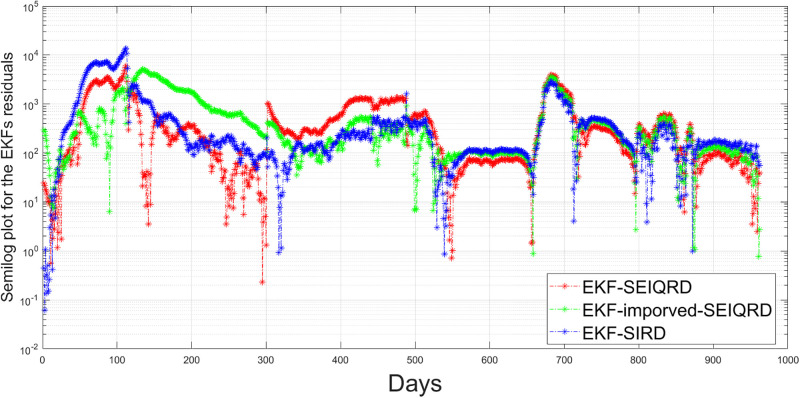
Comparison of residuals estimations for death cases: Assessing the EKF-SEIQRD, EKF-improved-SEIQRD, and EKF-SIRD models.

In this section, we compare the performance of the three models and discuss the implications of our results. Overall, the 6D models showed adequate estimation results with the time-varying mean posterior response by following the qualitative behaviour of the active cases data as shown in Fig 23. For the SIRD model estimation, we noticed that the model can follow the trajectory of the first wave but from the 400th day, the model yielded a biased estimate for the second peak. However, the SIRD model may be sufficient for modelling mid-term behaviour where the SIRD model on the 700th day, is not able to reach the infection peak in contrast to the SEIQRD model and the improved-SEIQRD model. For the death cases estimation, was analyzed through the three models, as presented in Fig 24. However, for the death cases, it is challenging to determine the best model among them since each model provides its own estimation result. Also, each model aims to capture and simulate the underlying behaviour differently. Table 12, provides the root mean square error (RMSE) values for the three models estimation, where it appears that the SEIQRD model exhibits the smallest error estimation for both active cases and deaths using the formula:

$$\begin{array}{c} {\text{RMSE}}_{I,D}=\sqrt{\frac{1}{n}\sum_{i=1}^{n}{\left(X_{\text{reported}\;I,D}-X_{\text{models},I,D}\right)}^{2}}, \end{array}$$ (43)

where *n* is the length of the reported data. However, this discrepancy implies potential limitations in these models’ ability to precisely predict the number of active cases and deaths. Using these deterministic models without the EKF estimation, it is not possible to follow precisely the reported data. These estimation results were enhanced with the EKF-SEIQRD, EKF-improved-SEIQRD and EKF-SIRD as shown for the active cases in Fig 23. It is noticeable that the EKF performances in the three models in Figs 23 and 24 are close to the reported data where EKF iteratively updates the estimates through the predictor-corrector step which helps to minimize the variations between the model’s predictions and the reported data scaled by the Kalman gain. Generally, the EKF predictions in Figs 23 and 24 are more reliable than the three deterministic models due to capturing the uncertainties as noise distributions. The comparative view of discrepancies of active cases is presented in Fig 25, and for death cases in Fig 26. These residuals represent the differences between the EKF algorithm estimation and actual values for active and death cases.

**Table 12:** The Root Mean Square Error (RMSE) between models and reported data.

Model	RMSE	
	Infected error	Death error
SEIQRD	**336.11**	**1313.1**
Improved-SEIQRD	374.80	1370.5
SIRD	343.97	1714.7

**Fig 27:**
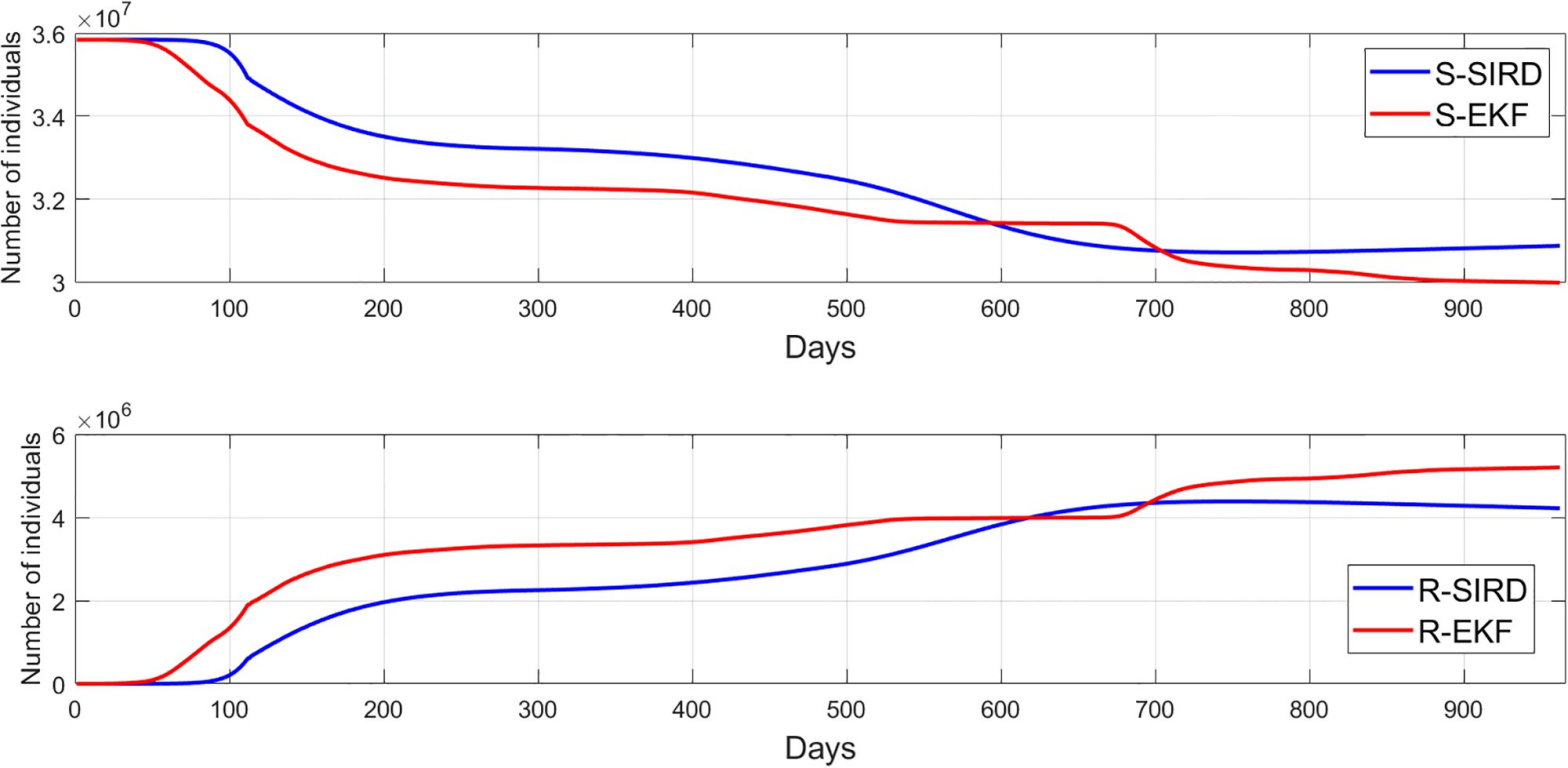
Comparison between the SIRD and SIRD-EKF models to estimate the unmeasurable states of susceptible and recovered compartments.

**Fig 28:**
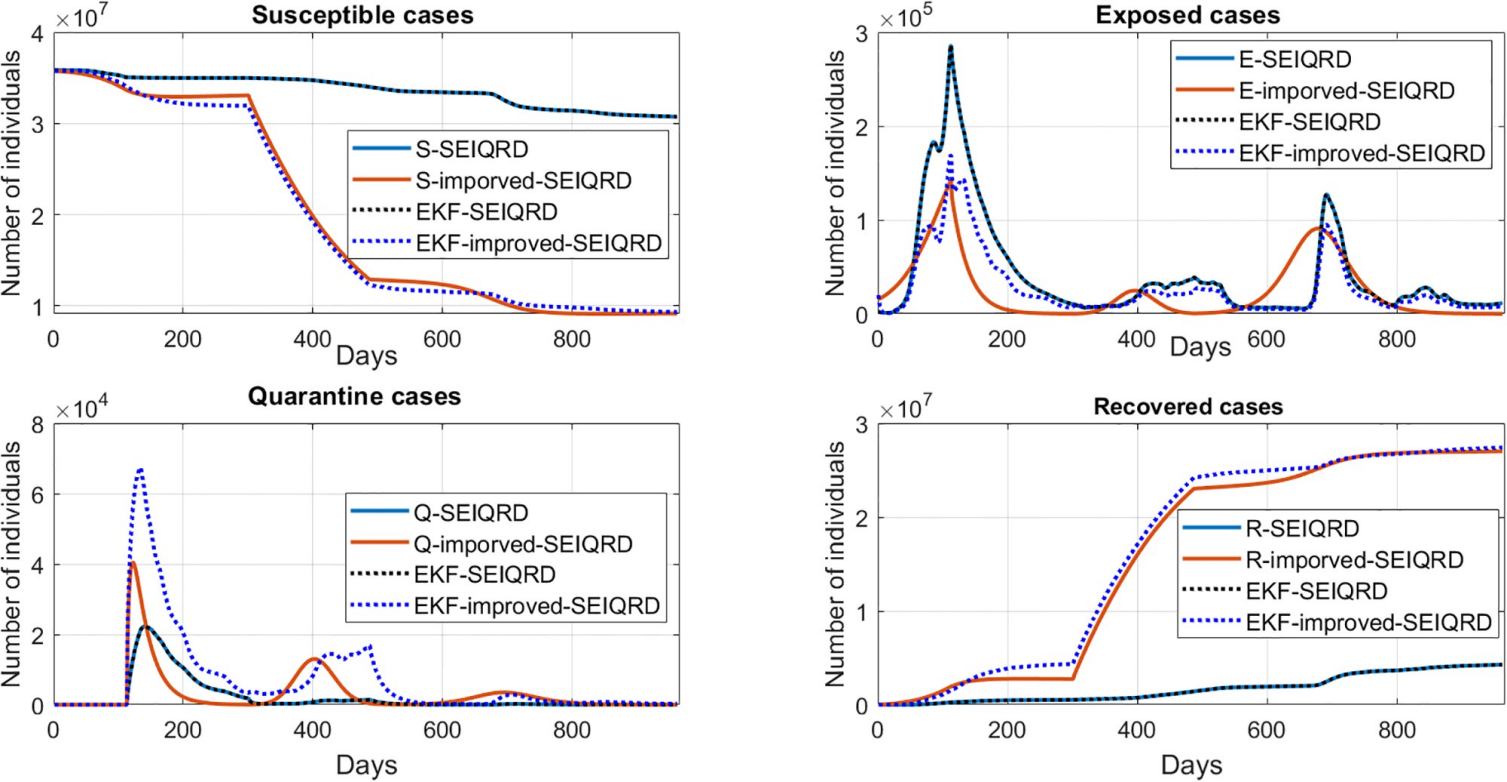
Comparison between the SEIQRD model and EKF-SEIQRD, improved-SEIQRD model, EKF-improved-SEIQRD to estimate the unmeasurable states of susceptible, exposed, quarantined and recovered cases.

**Fig 29:**
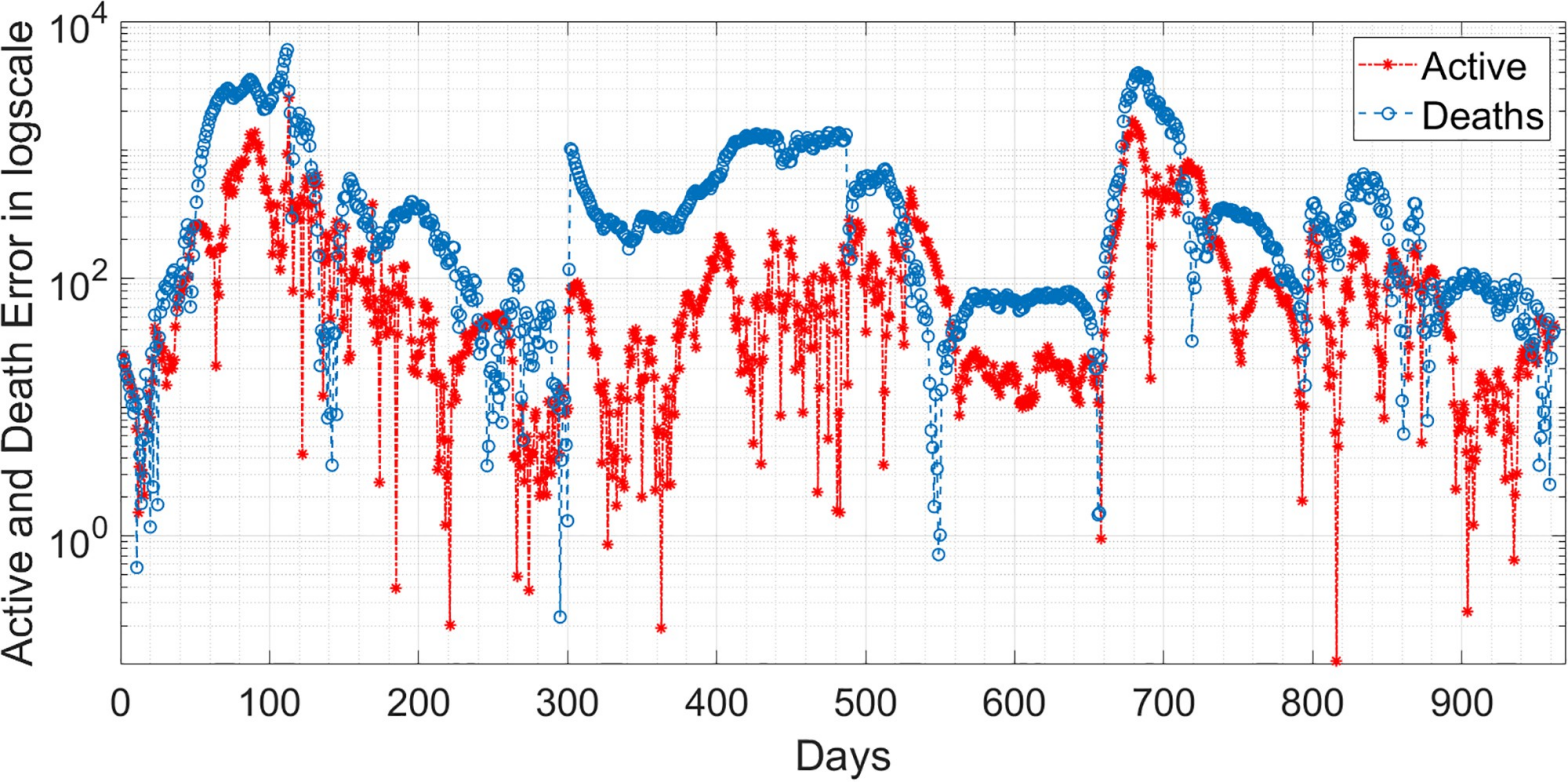
Semi-log plot of the estimation errors in active and death cases for the EKF-SEIQRD model.

**Fig 30:**
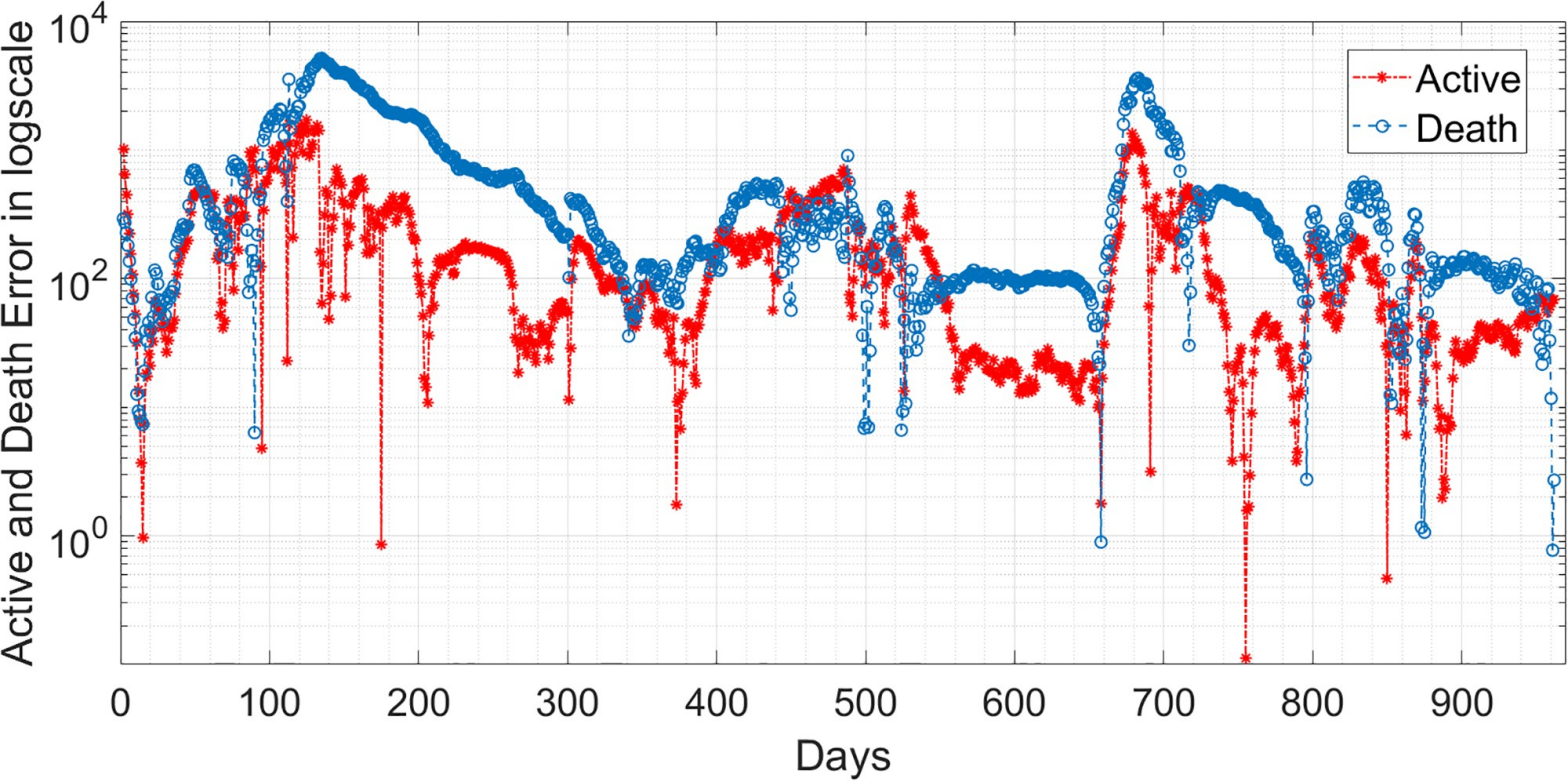
Semi-log plot of the estimation errors in active cases and death cases for the EKF-improved-SEIQRD model.

**Fig 31:**
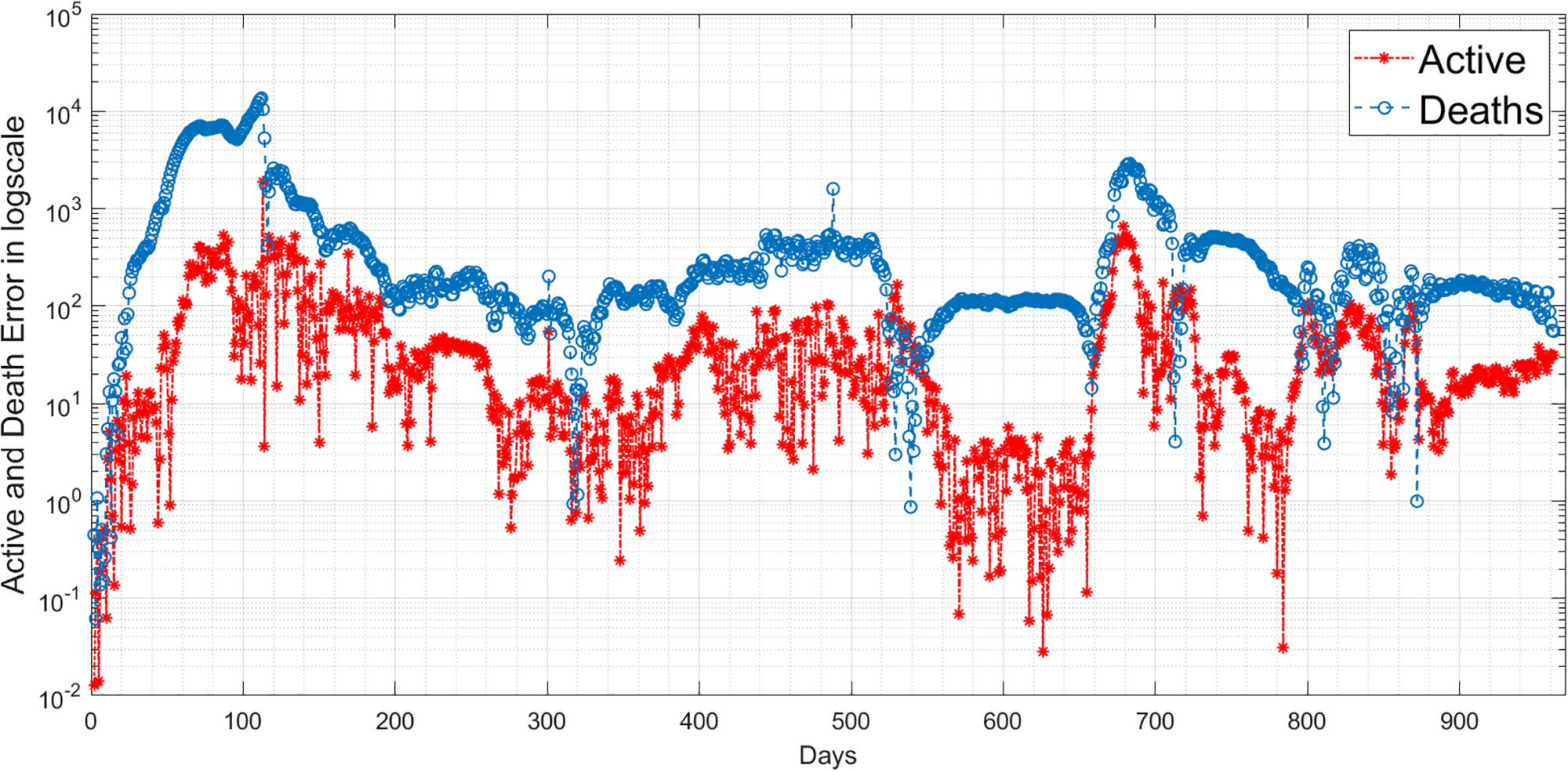
Semi-log plot of the estimation errors in active cases and death cases for the EKF-SIRD model.

Unmeasurable states of the SIRD model i.e. the susceptible cases *S* and recovered cases *R* are shown in Fig 27 as well as compared with the EKF estimation. The unmeasurable state estimates for the SEIQRD model and improved-SEIQRD which are the susceptible cases *S*, exposed cases *E*, quarantined cases *Q* and recovered cases *R* are compared using different EKFs performances as shown in Fig 28. From Fig 28, we noticed that the relaxation of quarantine and lockdown measures led to a flatness in the *Q* curve since less quarantining support to the return to normal life, as opposed to the early phase of the pandemic and there is a fluctuation in the number of exposed cases *E* which tried to follow the trajectory of the infected cases. For the susceptible cases and recovered cases in Fig 28, there is a difference between the EKF-SEIQRD performance and EKF-improved-SEIQRD performance. We can justify these deviations between EKFs performance to the effect of vaccination in the improved-SEIQRD model as they help minimize the number of susceptible and maximize the number of recovered as opposed to the SEIQRD model which doesn’t account for the vaccination parameter. Moreover, to evaluate the accuracy of the EKF predictions we use the RMSE to measure the difference between the (EKF-SEIQRD, EKF-improved-SEIQRD and EKF-SIRD) estimations and the reported data which is defined as:

$$\begin{array}{c} {\text{RMSE}}_{I,D}=\sqrt{\frac{1}{n}\sum_{i=1}^{n}{\left(X_{\text{reported}\;I,D}-X_{\text{EKFs},I,D}\right)}^{2}}, \end{array}$$ (44)

where *n* is the length of the reported data. Based on the RMSE values in Table 11, it seems that the performance of the two models in predicting the behaviour of COVID-19 is satisfactory. The lower RMSE for infected error with the EKF-SIRD indicates a closer model to observed data, but it exhibits larger errors in predicting death cases. This means that the EKF-SIRD model accurately captures the evolution of the active cases, but may have limitations in quantifying the dynamics of the death cases. For the 6D models, it seems the EKF-SEIQRD has lower RMSE for the infected and death error over the EKF-improved-SEIQRD model. For clear visualization of the infected and death error between the EKFs and observed data for the EKF, we analyse the semi-log plot for the EKF-SEIQRD model in Fig 29, for the EKF-improved-SEIQRD model in Fig 30 and the EKF-SIRD model in Fig 31. Here, we mainly show overall model comparison based on the average value of the Bayesian evidence since otherwise different models need to be used in different time segments. This will result in a switched dynamical system which is not considered in this analysis due to its increased complexity in stability and inclusion in the Kalman filtering methods. We have incorporated metrics to validate the accuracy of our simulation results for model predictions. Notably, the SEIQRD model demonstrates smaller errors, providing significant evidence of its efficacy in certain scenarios. Real-world epidemiological processes are highly complex and change with time due to the Government’s interventions. We here aim to identify the best model through Bayesian model selection followed by the accuracy of EKFs for prediction of the active and death cases. For other classes of more complex epidemiological models, the comparison may be extended and left as the scope for future research.

## 7 Conclusion

Different scenarios of unknown effects of parameter sensitivity of epidemiological models have been considered through the SEIQRD model and the improved-SEIQRD model. An approach for model comparison based on Bayesian inference and the EKF algorithm is presented for three epidemiological models: SEIQRD, improved-SEIQRD, and SIRD, in long-term behaviour. The nested sampling algorithm is utilized to obtain the posterior mean samples for the models’ parameters in a time-dependent context. Bayesian evidence for all the models is computed and used to compare between the three models. From a Bayesian inference perspective, the SEIQRD model outperforms the SIRD and improved-SEIQRD models, as evidenced by a higher log(*Z*) value, indicating a better fit to the data. The SIRD model exhibits a notably accurate estimate with a relatively mid-term behaviour, and more complex models may be beneficial for studying long-term behaviour and examining parameter sensitivity. Distinct estimation patterns were observed among the three models for the same dataset, as well as for the reproduction number $R_{0}$. This is due to inherent limitations and inadequacies in deterministic models, leading to biased estimates that impact the decision-making process. Another reason for this variation could be the utilization of different prior ranges for the three models, since using the same prior range was not applicable in this study. However, incorporating these models into the marginal likelihood, approximated by nested sampling within the EKF framework, could address the variability issues of epidemiological models. This approach goes beyond relying solely on epidemiological models, reducing model uncertainty and subsequently minimising the risks associated with the decision-making process. This confirms that the EKF algorithm demonstrates adaptability features, enhancing the accuracy of estimations across different epidemiological models. Consequently, the KF algorithms with their different extensions can be part of model validation, besides its main purpose in state estimation and prediction in dynamical systems. The simulation results of COVID-19 spread using the EKF-SEIQRD, EKF-improved-SEIQRD and EKF-SIRD, yielded accurate predictions of the number of active cases and deaths along with providing state estimation for the unmeasurable states based on the RMSE between the models estimation and reported data. Moreover, the tuning of the covariance measurement matrix was based on the method that was investigated in our previous approach in [76], which proved effective in reducing the estimation error. Finally, the approach in this study can be used for model validation regardless of the format of the data with different sources, models and populations which offers significant advantages in terms of accuracy, uncertainty quantification, adaptivity, and data dimensionality, which can be used for other possible future pandemics. To the best of our knowledge, this is the first study of model selection methods in such settings. In the future, we will use the EKF to solve the issue of the variations in the $R_{0}$. In addition, we shall consider more epidemiological model comparisons under the generalized Bayesian inference framework with other types of more advanced Kalman filter variants and state estimation algorithms with different noise distributions and higher order moments [103].
